# Cholesterol Depletion‐Enhanced Ferroptosis and Immunotherapy via Engineered Nanozyme

**DOI:** 10.1002/advs.202405826

**Published:** 2024-08-09

**Authors:** Tingjie Bai, Panpan Xue, Sijie Shao, Shuangqian Yan, Xuemei Zeng

**Affiliations:** ^1^ Key Laboratory of Microbial Pathogenesis and Interventions of Fujian Province University Biomedical Research Center of South China College of Life Sciences Fujian Normal University Fuzhou 350117 China; ^2^ The Straits Institute of Flexible Electronics (SIFE Future Technologies) Straits Laboratory of Flexible Electronics (SLoFE) Fujian Normal University Fuzhou 350117 China

**Keywords:** cholesterol metabolism, ferroptosis, immunotherapy, nanozyme

## Abstract

Ferroptosis, an iron‐ and reactive oxygen species (ROS)‐dependent cell death, holds significant promise for tumor therapy due to its ability to induce lipid peroxidation (LPO) and trigger antitumor immune responses. However, elevated cholesterol levels in cancer cells impede ferroptosis and compromise immune function. Here, a novel nanozyme, Fe‐MOF/CP, composed of iron metal‐organic framework (Fe‐MOF) nanoparticles loaded with cholesterol oxidase and PEGylation for integrated ferroptosis and immunotherapy is introduced. Fe‐MOF/CP depletes cholesterol and generates hydrogen peroxide, enhancing ROS levels and inducing LPO, thereby promoting ferroptosis. This process disrupts lipid raft integrity and downregulates glutathione peroxidase 4 and ferroptosis suppressor protein 1, further facilitating ferroptosis. Concurrently, Fe‐MOF/CP augments immunogenic cell death, reduces programmed death‐ligand 1 expression, and revitalizes exhausted CD8^+^ T cells. In vivo studies demonstrate significant therapeutic efficacy in abscopal, metastasis, and recurrent tumor models, highlighting the robust antitumor immune responses elicited by Fe‐MOF/CP. This study underscores the potential of Fe‐MOF/CP as a multifunctional therapeutic agent that combines ferroptosis and immunotherapy, offering a promising strategy for effective and durable cancer treatment.

## Graphical abstract

This work presents a hybrid nanozyme composed of PEGylated Fe‐MOF nanoparticles and cholesterol oxidase, termed Fe‐MOF/CP, designed to initiate combinatorial tumor ferroptosis immunotherapy. The findings indicate that cholesterol depletion not only enhances the catalytic efficiency of the nanozyme but also augments tumoral ferroptosis and antitumor immune responses, highlighting the significant potential of cholesterol modulation in cancer treatment.

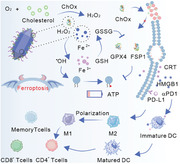

## 1 Introduction

Ferroptosis is a type of reactive oxygen species (ROS)‐ and iron‐dependent cell death, characterized by lethal lipid peroxidation (LPO) accumulation.^[^ ^1^ ^]^ Specifically, iron‐based Fenton reaction catalyzes tumoral H_2_O_2_ to ROS, such as hydroxyl radical (^•^OH), which oxidizes polyunsaturated fatty acids (PUFAs) in the cellular membrane, leading to membrane rupture and cell death.^[^ ^2^, ^3^ ^]^ Moreover, immunogenic ferroptotic cells can release danger‐associated molecular patterns (DAMPs), triggering antitumor immune responses that further enhance tumor ferroptosis and impede tumor metastasis.^[^ ^4^, ^5^, ^6^ ^]^ However, the limited ROS pool and antioxidative substrates like glutathione (GSH) within cancer cells counteract ferroptosis.^[^ ^7^, ^8^ ^]^ Additionally, cell‐intrinsic anti‐ferroptosis proteins, such as glutathione peroxidase 4 (GPX4) and ferroptosis suppressor protein 1 (FSP1), detoxify lipid peroxides, thereby suppressing ferroptosis.^[^ ^9^, ^10^, ^11^ ^]^ Furthermore, ferroptosis‐activated immune cells often face reprogramming by the immune‐suppressive microenvironment.^[^ ^12^ ^]^ Therefore, overcoming these challenges is crucial to enhancing therapeutic efficacy by amplifying cell ferroptosis and revitalizing ferroptosis‐associated antitumor immune responses.

Cholesterol, an indispensable structural component of membranes, plays a crucial role in maintaining membrane integrity and fluidity.^[^ ^13^ ^]^ In cancer cells, the heightened uptake via up‐regulation of lipoprotein receptors and synthesis rates through the hyperactive mevalonate pathway result in cholesterol accumulation, fostering cellular proliferation, tumor metastasis, and immune suppression.^[^ ^14^, ^15^ ^]^ Recent studies have revealed that GPX4 and FSP1 rely on outputs from the mevalonate pathway to hinder LPO generation and sensitivity to ferroptosis.^[^ ^16^, ^17^, ^18^ ^]^ Elevated cholesterol levels reduce membrane fluidity and facilitate lipid raft formation, thereby inhibiting ferroptosis by impeding the diffusion of LPO substrates.^[^ ^19^, ^20^ ^]^ Moreover, cholesterol intermediate metabolites like 7‐dehydrocholesterol and oxidative by‐products such as 27‐hydroxycholesterol have been demonstrated to suppress ferroptosis.^[^ ^21^, ^22^ ^]^ Notably, cholesterol contributes to tumor immunosuppression in complex ways. For instance, it can upregulate the expression of programmed‐death ligand 1 (PD‐L1), facilitating immune evasion in cancer cells.^[^ ^23^ ^]^ Furthermore, cholesterol induces CD8^+^ T cell exhaustion via an endoplasmic reticulum stress‐XBP1‐dependent mechanism.^[^ ^24^ ^]^ Consequently, reducing cholesterol in the tumor microenvironment not only sensitizes cellular ferroptosis but also revitalizes T cell function and reverses tumor immunosuppression,^[^ ^25^ ^]^ highlighting its significant potential in tumor synergistic ferroptosis‐immune therapy.

Biological enzymes exhibit potent catalytic activity, remarkable specificity, and excellent biocompatibility, making them valuable tools in tumor therapy by target depletion.^[^ ^26^, ^27^, ^28^ ^]^ Cholesterol oxidase (ChOx), which catalyzes cholesterol to H_2_O_2_ and cholestenone,^[^ ^29^, ^30^, ^31^, ^32^, ^33^ ^]^ is a promising candidate for enhancing ferroptosis‐immune therapy through cholesterol depletion. However, similar to small molecular drugs, ChOx faces challenges such as poor pharmacokinetics, inadequate tumor targeting, and off‐target side effects.^[^ ^34^ ^]^ In recent years, nanoparticles have emerged as a solution to overcome these limitations associated with free drugs in tumor treatment.^[^ ^35^ ^]^ Additionally, nanoparticles with biological enzyme‐mimicking properties, known as nanozymes,^[^ ^36^, ^37^ ^]^ have been utilized to induce ferroptosis by increasing the pool of ROS.^[^ ^38^, ^39^, ^40^, ^41^, ^42^, ^43^, ^44^, ^45^, ^46^, ^47^ ^]^ Therefore, we hypothesize that combining ferroptosis‐activable nanozymes with ChOx will result in significant therapeutic efficacy.

**Scheme 1:**
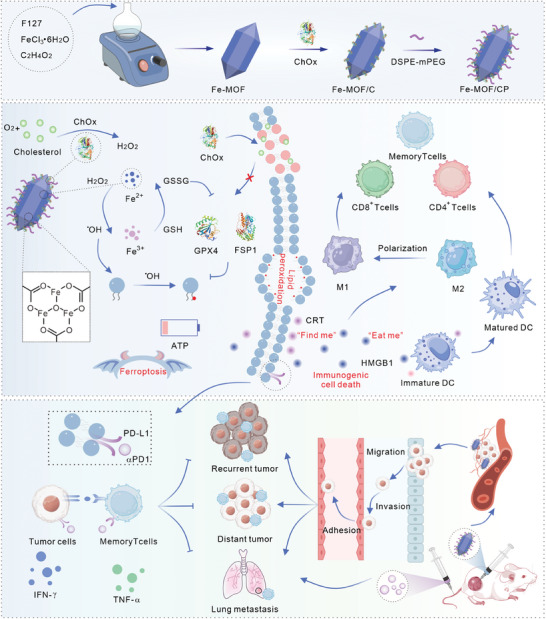
Preparation scheme of Fe‐MOF/CP hybrid nanozyme and the proposed mechanism of cholesterol‐depleting Fe‐MOF/CP for tumor synergistic ferroptosis‐immune therapy.

In this study, we introduce a hybrid nanozyme, termed Fe‐MOF/CP, composed of iron metal‐organic framework (Fe‐MOF) nanoparticles, biological ChOx, and PEGylation, for integrated ferroptosis‐immune tumor therapy. The Fe‐MOF nanoparticle exhibits peroxidase (POD)‐like activity, catalyzing tumoral H_2_O_2_ to ^•^OH, thereby amplifying ROS levels and inducing ferroptosis in cancer cells. Additionally, ChOx is released from Fe‐MOF/CP in a pH‐responsive manner, leading to cholesterol depletion and H_2_O_2_ generation, further enhancing cellular ROS levels. We observe that Fe‐MOF/CP downregulates the expression of GPX4 and FSP1 by disrupting cellular lipid rafts, thereby enhancing ferroptosis. Moreover, ChOx promotes immunogenic cell death and reduces PD‐L1 expression in cancer cells treated with Fe‐MOF nanoparticles. In vivo experiments confirm that Fe‐MOF/CP induces robust antitumor immune responses, including dendritic cell (DC) maturation, M2 macrophage polarization, attenuation of CD8^+^ T cell exhaustion, and activation of effector memory T cells. Notably, Fe‐MOF/CP hybrid nanozyme demonstrates significant therapeutic efficacy in abscopal, metastasis, and recurrent mouse models (**Scheme** **1**).

## 2 Results and Discussion

### 2.1 Fabrication and Characterization of Fe‐MOF/CP

**Figure 1:**
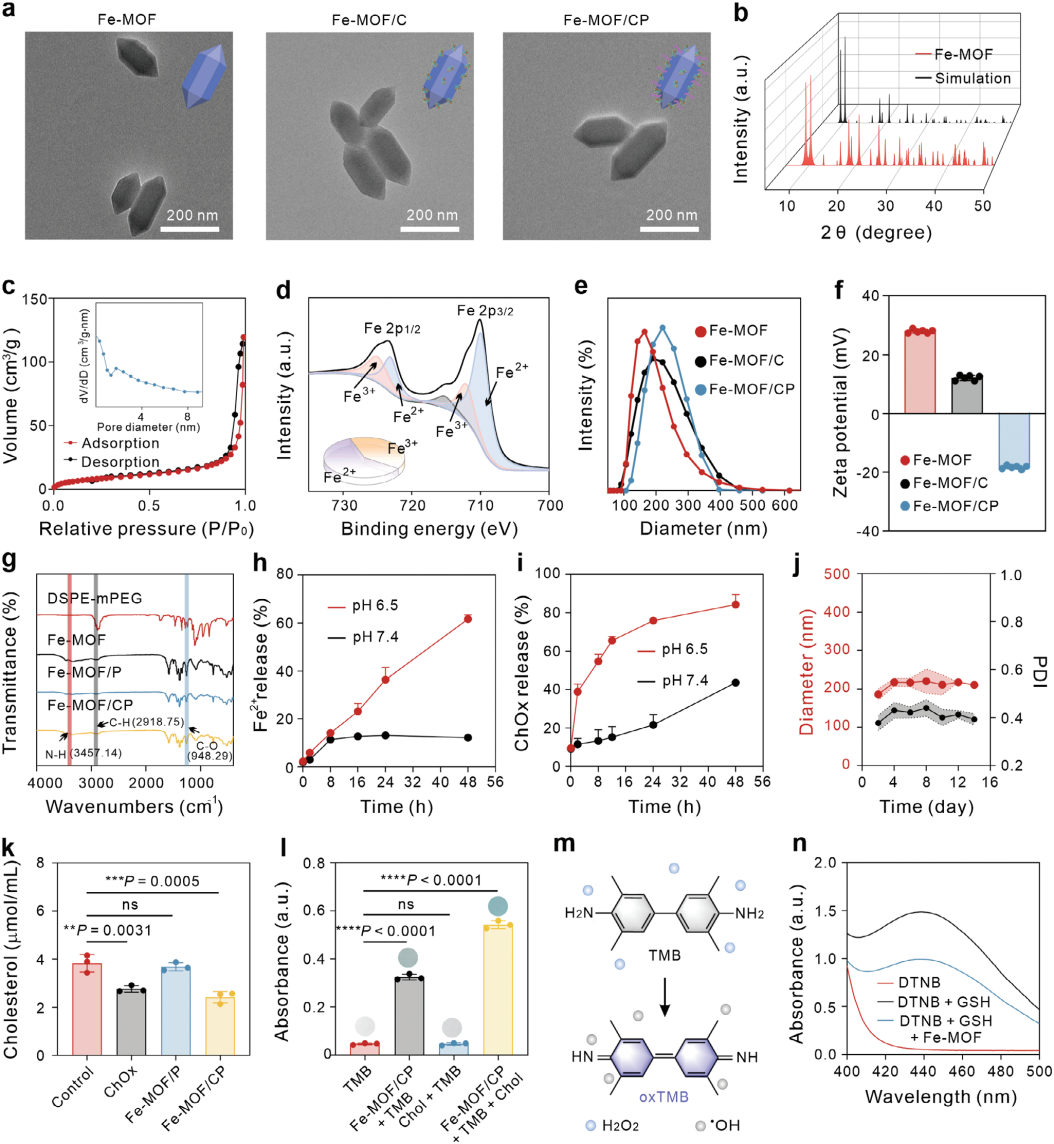
Characterization of Fe‐MOF/CP nanoparticles. a) TEM images depicting Fe‐MOF, Fe‐MOF/C, and Fe‐MOF/CP. b) XRD pattern of Fe‐MOF and simulation pattern. c) N_2_ adsorption/desorption isotherms of Fe‐MOF. d) High‐resolution Fe 2p spectra of Fe‐MOF. Insert: Percentage of Fe^2+^ and Fe^3+^ in Fe‐MOF. e) Hydrodynamic diameters of Fe‐MOF during various modifications. f) Zeta potentials of Fe‐MOF during various modifications, *n* = 3, represent 3 biological samples. g) FTIR spectra of PEG, Fe‐MOF, Fe‐MOF/C, and Fe‐MOF/CP. h,i) Release profiles of Fe^2+^ h) and ChOx i) from Fe‐MOF/CP under pH 6.5 and 7.4 conditions, *n* = 3. j) Colloidal stability of Fe‐MOF/CP in PBS (pH 7.4) containing 10% fetal bovine serum at 4 °C, *n* = 3. k) Concentration of cholesterol after treatment with various formulations, *n* = 3. l) The absorption intensity of colored oxTMB at 650 nm, *n* = 3. Cholesterol was denoted as Chol. m) Schematic illustration of TMB oxidation by ^•^OH. n) UV–Vis spectra of DTNB solutions after reactions. Statistical significance denoted as **p < 0.01, ***p < 0.001, ****p < 0.0001, and ns: not significant (*p* > 0.05), analyzed by one‐way ANOVA, followed by Dunnett's multiple comparisons test. Data represent mean ± standard deviation (s.d.).

Fe‐MOF nanoparticles were synthesized according to our previously reported method.^[^ ^41^ ^]^ Transmission electron microscopy (TEM) images revealed monodispersed Fe‐MOF nanoparticles with a shuttle shape, measuring 180 nm in length and 50 nm in width (**Figure** **1**a). X‐ray diffraction (XRD) spectra exhibited a well‐defined crystalline structure for the Fe‐MOF nanoparticles, matching stimulated patterns (Figure 1b). Brunner‐Emmet‐Teller (BET) measurements indicated a surface area of 4.3182 m^2^g^−1^ and an average pore diameter of ≈2.7 nm (Figure 1c). X‐ray photoelectron spectroscopy (XPS) analysis surveyed the element composition of synthesized Fe‐MOF nanoparticles (Figure S1, Supporting Information) and the valence state of iron ions (Figure 1d). We found that Fe 2p spectrum displayed peaks at ≈723.48 and 710.28 eV, corresponding to Fe 2p1/2 and Fe 2p3/2, respectively. Moreover, peak‐differentiating revealed a mixed valence of Fe^2+^ and Fe^3+^ in Fe‐MOF.^[^ ^48^ ^]^ ChOx and/or DSPE‐mPEG were then mixed with Fe‐MOF to obtain Fe‐MOF/C and Fe‐MOF/CP. Morphologically, both Fe‐MOF/C and Fe‐MOF/CP showed no differences compared to Fe‐MOF (Figure 1a), indicating that ChOx loading or PEGylation did not affect the nanoparticle morphology. Dynamic light scattering analysis revealed hydrodynamic sizes of 164 nm for Fe‐MOF, 190 nm for Fe‐MOF/C, and 220 nm for Fe‐MOF/CP (Figure 1e). Zeta potential measurements showed a positive charge of 28.0 mV for Fe‐MOF nanoparticles (Figure 1f), facilitating negative‐charged therapeutic loading. After ChOx encapsulation, the potential of Fe‐MOF/C was reduced to 12.1 mV. The negative charge of Fe‐MOF/CP is attributed to the large number of methoxy groups in DSPE‐mPEG. UV–vis spectra analysis provided evidence of ChOx loaded in Fe‐MOF nanoparticles (Figure S2, Supporting Information). As can be seen from Fourier transformed infrared (FTIR) spectra in Figure 1g, the emerging peaks at 2457.14 cm^−1^ in Fe‐MOF/C and at 2918.75 and 948.29 cm^−1^ in Fe‐MOF/CP, corresponding to N‐H groups in ChOx and C‐H and C‐O groups in DSPE‐mPEG molecules, respectively. Collectively, these results validated the successful construction of Fe‐MOF/CP, with ChOx loading efficacy calculated at 60 µg ChOx per 100 µg Fe‐MOF via a nanodrop measurement. Besides, the results of the BCA measurement kit further verified the loading rate was 62.8% (Table S1, Supporting Information).

Next, we investigated the degradation and therapeutic release kinetics of the Fe‐MOF/CP nanozyme under both neutral and moderately acidic conditions. Our observations revealed that Fe‐MOF/CP remains stable in pH 7.4 solution (Figure S3, Supporting Information). However, degradation occurred in the presence of glutathione (GSH). Notably, under pH 6.5, the morphology of Fe‐MOF/CP was disrupted, with this effect further amplified upon GSH treatment, indicating pH/GSH‐responsive degradability. As depicted in Figure 1h, the release of Fe^2+^ from Fe‐MOF/CP amounted to only 12.2% within 48 h in pH 7.4 PBS solution at 37 °C, as determined by the Fe^2+^ standard curve (Figure S4, Supporting Information). In contrast, the release of Fe^2+^ was notably accelerated in the pH 6.5 buffer solution, reaching a release ratio of 61.6% at 48 h. Similarly, Fe‐MOF/CP exhibited higher ChOx release profiles under acidic conditions compared to neutral circumstances (Figures 1i and S5, Supporting Information). These findings provide additional evidence of the pH‐responsive nature of Fe‐MOF/CP, suggesting its potential for tumor‐specific therapeutic release and therapy. Additionally, the as‐prepared Fe‐MOF/CP nanozyme showed negligible fluctuation in hydrodynamic size and polydispersity index during storage in PBS supplemented with 10% fetal bovine serum for 2 weeks at 4 °C (Figure 1j), indicating good storage stability.

Subsequently, we assessed the cholesterol‐depleting capability of Fe‐MOF/CP. Initially, Fe‐MOF/CP and free ChOx were first incubated with cholesterol, and the cholesterol content was quantified using a standard curve based on a commercial kit (Figure S6, Supporting Information). As illustrated in Figure 1k, both ChOx and Fe‐MOF/CP reduced the concentration of cholesterol compared to the control group, demonstrating the cholesterol consumption capacity of the Fe‐MOF/CP nanozyme. The robust activity of the enzyme is crucial for its extensive applications. To further validate the activity of Fe‐MOF/CP, we simulated high‐temperature and acidic‐basic environments to assess its catalytic ability to degrade cholesterol. As shown in Figure S7 (Supporting Information), Fe‐MOF/CP exhibited substantial stability at elevated temperatures and varying pH levels, in contrast to free ChOx, which demonstrated significantly reduced activity under the same conditions. This indicates that the Fe‐MOF framework effectively enhances the stability and functionality of ChOx. Next, we investigated the characteristics of Fe‐MOF as a nanozyme and visualized its POD‐like and GSHox‐like activities (Figure S8, Supporting Information). According to electron paramagnetic resonance (EPR) spectroscopy, Fe‐MOF generates ^•^OH radicals upon reaction with H_2_O_2_ (Figure S9, Supporting Information). Moreover, the ^•^OH generation of Fe‐MOF by Fenton‐like reaction was assessed by tetramethylbenzidine (TMB), which oxidizes collarless TMB to blue‐colored oxTMB. As can be seen from Figure S10 (Supporting Information), Fe‐MOF nanoparticles oxidated TMB in the presence of H_2_O_2_. Similar outcomes were observed with o‐phenylenediamine (OPD) chromogenic reaction (Figure S11, Supporting Information) and methylene blue (MB) degradation assays (Figure S12). The POD‐like enzyme activity of Fe‐MOF followed Michaelis‐Menten kinetics, yielding a K_m_ of 11.25 mM and a V_max_ of 1.24 × 10^−7^ M min^−1^. (Figure S13, Supporting Information). Additionally, Fe‐MOF nanoparticles exhibited superior catalytic potency in acidic solutions compared to neutral solutions (Figure S14, Supporting Information). These results underscore the outstanding ^•^OH generation capacity of Fe‐MOF, suggesting its potential for tumor‐specific ROS elevation. Functionally, ChOx can produce H_2_O_2_ by oxidizing cholesterol, thereby enhancing the POD activity of Fe‐MOF, as confirmed by the TMB chromogenic reaction (Figure 1l,m). Specifically, Fe‐MOF also exhibited GSH oxidase‐mimicking activity, as evidenced by 5,5′‐dithio‐bis(2‐nitrobenzoic acid) (DTNB) absorption assay (Figures 1n and S15, Supporting Information), which contributes to the elevation of ROS pool and ferroptosis. This was further corroborated by XPS spectroscopy (Figure S16, Supporting Information), which showed that the proportion of Fe^3+^ declined by 37.2% (from 39.2% to 2%) after treatment with GSH. Furthermore, the aforementioned reaction also adhered to Michaelis‐Menten kinetics, exhibiting K_m_ and V_max_ values of 6.7 mM and 8.8 × 10^−8^ M min^−1^, respectively (Figure S17, Supporting Information).

### 2.2 Fe‐MOF/CP Depletes Cholesterol in Cancer Cells

**Figure 2:**
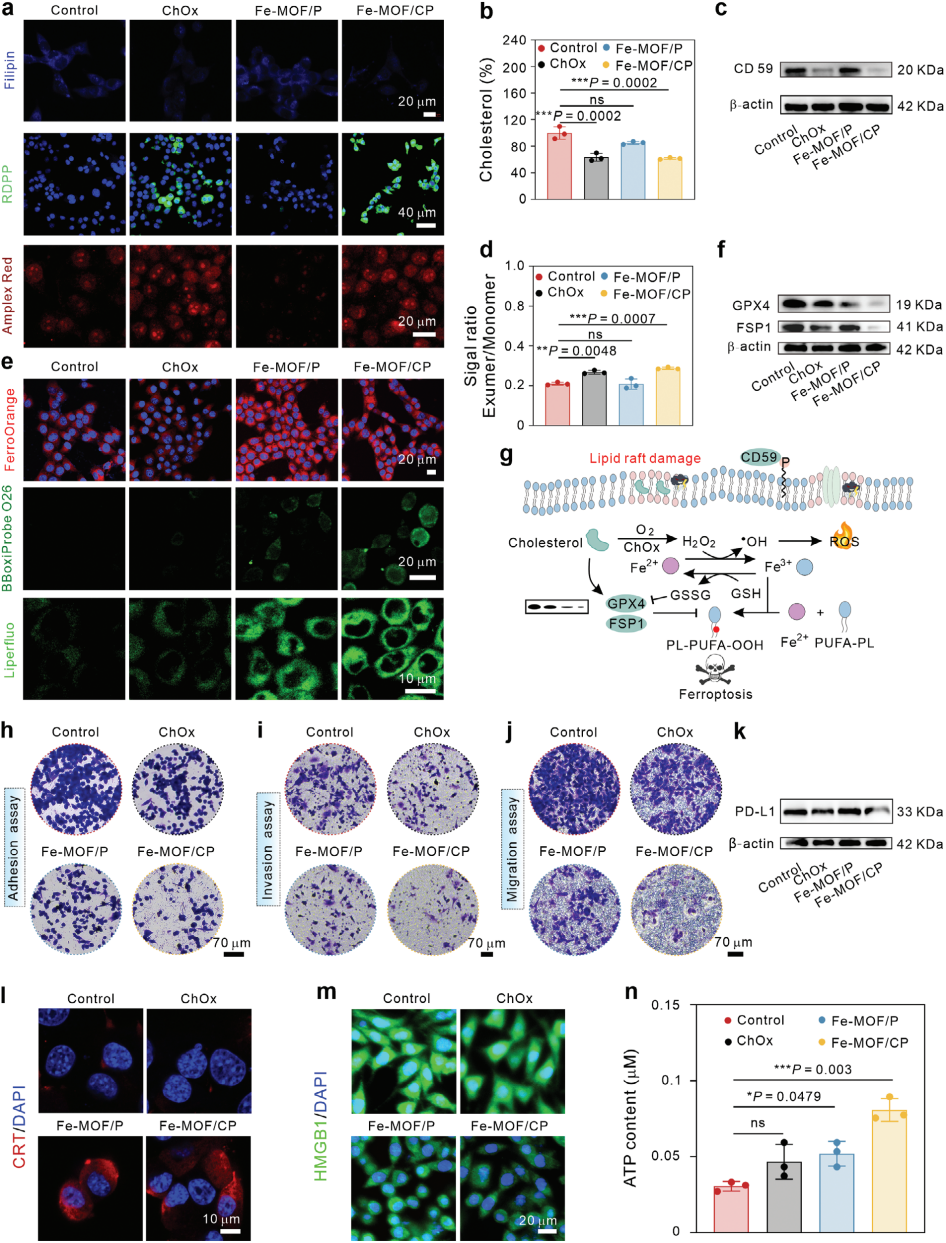
Fe‐MOF/CP enhances cellular ferroptosis and ICD responses. a) Cellular fluorescent images of 4T1 cells under indicated treatments. Filipin: cholesterol; RDPP: oxygen; and Amplex Red: H_2_O_2_. b) Intracellular cholesterol percentages in 4T1 cells treated with different formulations, *n* = 3. c) Western blotting analysis of the expression of CD59 in 4T1 cells after different treatments. d) Cell membrane fluidity of 4T1 cells with indicated treatments, *n* = 3. e) Fluorescence staining of Fe^2+^ (FerroOrange), •OH (BBoxiProbe O26), and LPO (Liperfluo). Blue indicates the cell nucleus. f) Expression of GPX4 and FSP1. g) The schematic diagram of cholesterol depletion by Fe‐MOF/CP for ferroptosis activation. h–j) Photographs of migration (h), adhesion (i), and invasion (j) of 4T1 cells with indicated treatments. Scale bars are 70 µm. k) PD‐L1 expression in 4T1 cells upon various treatments. l,m) Fluorescent images of CRT l) and HMGB1 m) in 4T1 cells. n) ATP from the supernatant of 4T1 cells with indicated treatments, *n* = 3. Statistical significance denoted as **p < 0.01, ***p < 0.001, ****p < 0.0001, and ns: not significant (*p* > 0.05), analyzed by one‐way ANOVA, followed by Dunnett's multiple comparisons test. Data represent mean ± s.d.

The mouse breast cancer cell line 4T1 was employed in this study due to its intracellular cholesterol accumulation and formation of immunosuppressive tumors. 4T1 cells were incubated with fluorescein (FITC)‐labeled Fe‐MOF/P (Fe‐MOF/P@FITC), and the intracellular fluorescent intensity was measured using flow cytometric measurement (FCM). As shown in Figure S18 (Supporting Information), intracellular fluorescent intensity significantly increased after only 1 h incubation and continued to rise over time, indicating the abundant uptake of Fe‐MOF/P by 4T1 cells. Next, we investigated the cholesterol depletion ability of Fe‐MOF/CP. Filipin complex is a fluorescent dye that specifically binds to cholesterol. Therefore, the cholesterol content was labeled with Filipin complex and visualized using confocal laser scanning microscopy (CLSM). According to **Figures** **2**a and S19 (Supporting Information), cells treated with Fe‐MOF/P showed no obvious changes in blue fluorescence signals compared to the control group, while cells treated with ChOx and Fe‐MOF/CP displayed diminished blue signals. Furthermore, the cholesterol content in ChOx and Fe‐MOF/CP treated cells significantly decreased based on quantitative analysis using a commercial kit (Figure 2b). ChOx catalyzes O_2_ and cholesterol to H_2_O_2_ and cholestenone. Thus, we investigated the O_2_ and H_2_O_2_ levels in 4T1 cells using Ru(dpp)_3_]Cl_2_) (RDPP) and Amplex Red fluorescence probes, respectively. As depicted in Figures 2a and S20 (Supporting Information), there were no notable green RDPP fluorescence signals in control and Fe‐MOF/P‐treated cells, implying that Fe‐MOF/P has negligible effects on cellular O_2_. In contrast, ChOx and Fe‐MOF/CP‐treated cells displayed strong signals, demonstrating their ability to decrease O_2_ in cancer cells. Additionally, both ChOx and Fe‐MOF/CP increased the cellular Amplex Red fluorescence intensity, indicating an elevation in H_2_O_2_ concentration (Figures 2a and S21, Supporting Information). These findings demonstrate the efficient cholesterol depletion capacity of Fe‐MOF/CP in cancer cells. Afterward, we evaluated the lipid rafts of 4T1 cells after cholesterol depletion by analyzing the expression of CD59, a marker of lipid raft generation. As shown in Figures 2c and S22 (Supporting Information), both ChOx and Fe‐MOF/CP reduced the CD59 in cells. Besides, the results shown in Figures 2d and S23 (Supporting Information) implied that ChOx and Fe‐MOF/CP increased the membrane fluidity in 4T1 cells. Collectively, Fe‐MOF/CP can deplete cellular cholesterol, leading to H_2_O_2_ generation, lipid raft destruction, and membrane fluidity elevation in cancer cells.

### 2.3 Cholesterol Depletion by Fe‐MOF/CP Enhances Ferroptosis

As can be seen from Figure S24 (Supporting Information), both Fe‐MOF/P and Fe‐MOF/CP reduced the levels of GSH in cancer cells due to their GSH oxidase‐like activities. Besides, the intracellular GSH levels were assessed by staining cells with bromodiamine, a GSH fluorescent dye. As shown in Figure S25 (Supporting Information), both Fe‐MOF/P and Fe‐MOF/CP exhibited faint blue fluorescence, indicating their GSH oxidase‐like activity. Additionally, Fe‐MOF/CP increased cellular Fe^2+^ levels, as evidenced by FerroOrange staining (Figure 2e) and the corresponding quantitative analysis (Figure S26, Supporting Information). The POD‐like activity of Fe‐MOF/P and the released Fe^2+^ could catalyze cellular H_2_O_2_ to ^•^OH, thereby augmenting the pool of lethal ROS (Figures 2e and S27, Supporting Information). We found that Fe‐MOF/CP‐treated cells exhibited higher fluorescence intensity than Fe‐MOF/P‐treated cells, directly attributed to elevated H_2_O_2_ levels induced by ChOx. As depicted in Figures 2e and S28 (Supporting Information), Fe‐MOF/CP‐treated cells had higher fluorescence signals than those treated with ChOx alone and Fe‐MOF/P, indicating the superior ability of Fe‐MOF/CP to induce cellular LPO generation and ferroptosis compared to Fe‐MOF/P without ChOx loading. The flow cytometric results in Figure S29 (Supporting Information) corroborated the CLSM images. Compared to free ChOx (15.7%) and Fe‐MOF (64.4%), Fe‐MOF/CP (73.1%) induced a significantly higher percentage of LPOs in 4T1 cells, robustly confirming its profound effect on LPO generation. Additionally, 4‐hydroxynonenal (4‐HNE), a secondary product of LPO, was employed to quantify LPO levels in cells. Western blot analysis (Figure S30, Supporting Information) showed that Fe‐MOF/CP‐treated cells exhibited higher 4‐HNE expression compared to other groups, further confirming that Fe‐MOF/CP induces ferroptosis. Next, the impact of cholesterol depletion on ferroptosis suppressor proteins, including GPX4 and FSP1, was investigated by western blot (Figures 2f and S31, Supporting Information). We found that ChOx decreased the expression of GPX4 and FSP1, and Fe‐MOF/P suppressed GPX4 through GSH consumption. Notably, Fe‐MOF/CP significantly down‐regulated the GPX4 and FSP1, providing additional evidence of the synergistic effect between ChOx and Fe‐MOF on ferroptosis activation. The present results suggest that Fe‐MOF/CP induces pronounced cellular ferroptosis through ROS elevation and inhibition of anti‐ferroptosis proteins via cholesterol depletion (Figure 2g).

### 2.4 Fe‐MOF/CP Eradicates Cancer Cells and Triggers Immunogenic Cell Death

The in vitro therapeutic efficacy of Fe‐MPF/CP was assessed using the standard CCK‐8 assay. As depicted in Figure S32 (Supporting Information), Fe‐MOF/CP reduced 4T1 viability in a concentration‐dependent manner. Importantly, Fe‐MOF/CP at the same concentrations induced negligible apoptosis in T cells, demonstrating its favorable biocompatibility with CTLL2 cells (Figure S33, Supporting Information). Moreover, live/dead staining revealed that Fe‐MPF/CP induced extensive cell death (Figures S34, Supporting Information). It is widely acknowledged been well‐documented that cholesterol accumulation enhances cancer cell migration and invasion abilities, promoting tumor metastasis. Hence, depleting cholesterol by Fe‐MOF/CP may hinder cellular migration and invasion. Subsequently, we conducted cell migration and adhesion assays to evaluate the metastasis‐inhibiting effects of Fe‐MOF/CP. As illustrated in Figures 2h and S35 (Supporting Information), the migration ratios of cells treated with ChOx, Fe‐MOF/P, and Fe‐MOF/CP decreased to 66.2%, 42.7%, and 18.5%, respectively. Similar results were observed in cell adhesion assays (Figures 2i and S36, Supporting Information), with adhesion ratios of 59.7% for ChOx, 55.5% for Fe‐MOF/P, and 17.8% for Fe‐MOF/CP, suggesting that Fe‐MOF/CP impeded cell migration and adhesion. Additionally, we conducted a cell invasion assay to evaluate the motility of 4T1 cells following treatment (Figures 2k and S37, Supporting Information). The cell invasion percentages were 61.4% for ChOx, 61.6% for Fe‐MOF/P, and 39.6% for Fe‐MOF/CP. Similarly, Fe‐MOF/CP exhibited superior wound healing inhibition efficacy compared to ChOx alone and Fe‐MOF/P (Figure S38, Supporting Information). These findings suggest that the synthesized Fe–MOF/CP holds significant potential in hindering metastasis process of cancer cells.

Inspired by the role of cholesterol depletion in facilitating ferroptosis, we evaluated the in vitro immunogenic cell death (ICD) responses induced by Fe‐MOF/CP. As shown in Figures 2l and S39 (Supporting Information), ChOx did not induce the exposure of calreticulin (CRT), whereas Fe‐MOF/P exposed CRT, emanating from the immunogenic nature of the ferroptosis. Notably, Fe‐MOF/CP triggered more CRT exposure than Fe‐MOF/P. Moreover, Fe‐MOF/CP enhanced ATP secretion (Figures 2m and S40, Supporting Information) and high mobility group box 1 (HMGB1) release in 4T1 cells (Figures 2n andS41, Supporting Information). Specifically, Fe‐MOF/CP decreased the expression of PD‐L1 in cancer cells (Figures 2k and S42, Supporting Information). These results are consistent with the notion that cholesterol depletion not only enhances ferroptosis but also augments the ICD responses of Fe‐MOF/P nanoparticles.

### 2.5 Fe‐MOF/CP Depletes Tumoral Cholesterol and Induces Mouse Antitumor Immune Responses

**Figure 3:**
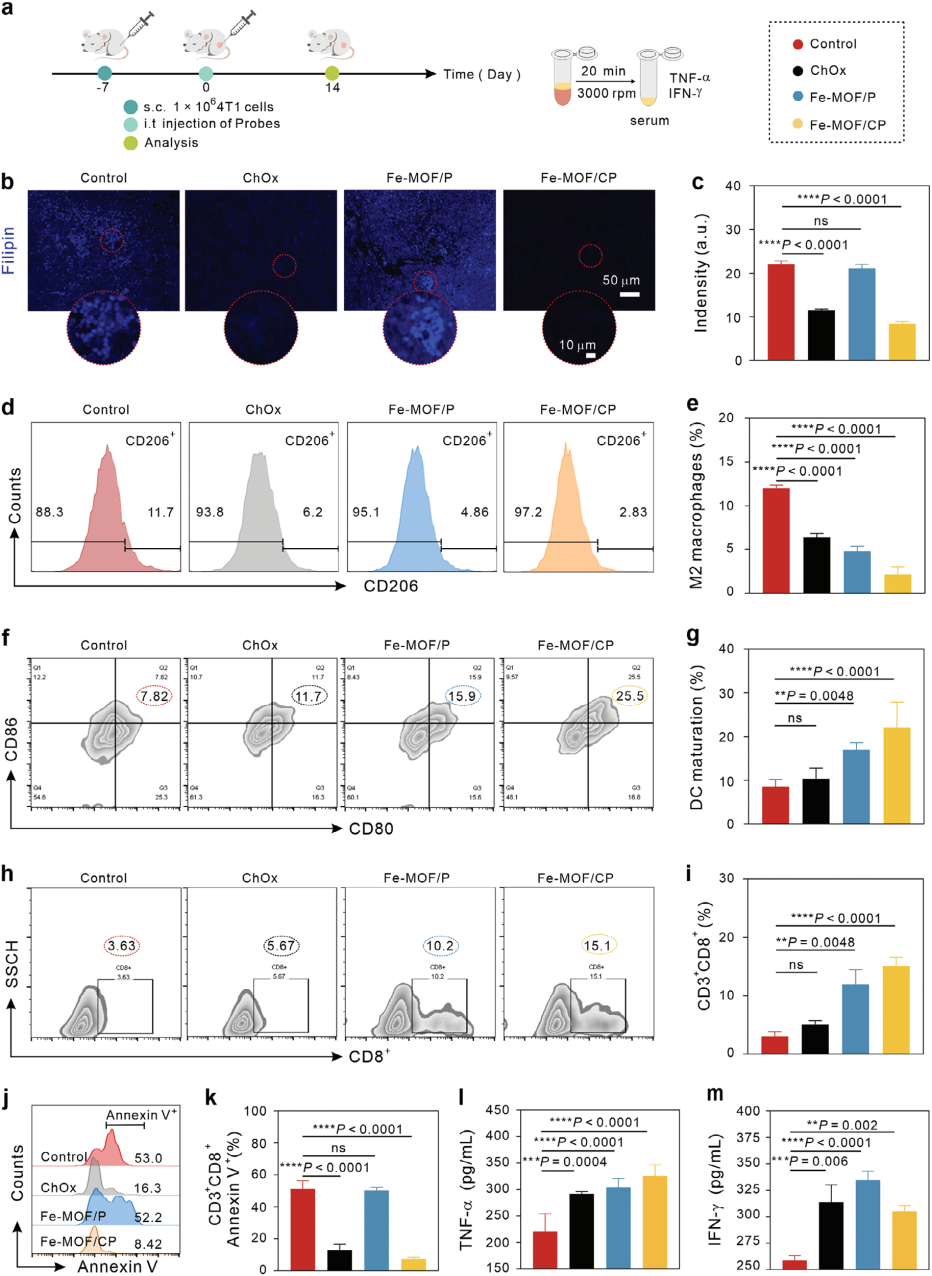
In vivo cholesterol depletion and immune response of Fe‐MOF/CP, *n* = 5. a) Treatment procedure. b,c) Fluorescent images (b) and corresponding statistical fluorescence intensity (c) of Filipin from tumor slices subjected to various treatments. d,e) Representative flow cytometric outcomes (d) and the percentage of M2 macrophages (gated on CD11b^+^F4/80^+^CD206^+^) (e) within tumors. f,g) Representative flow cytometric plots (f) and associated statistical results (g) indicating DC maturation (gated on CD11c^+^CD80^+^CD86^+^) in lymph nodes. h,i) Representative flow cytometric plots (h) and the percentage of CD8^+^ T cells (i) in tumors. j,k) Representative flow cytometric plots (j) and corresponding statistical results (k) of CD8^+^ T cells (gated on CD3^+^CD8^+^Annexin V^+^) within tumors. l,m) Measurement of TNF‐α (l) and IFN‐γ (m) levels in serum from mice subjected to indicated treatments. Statistical significance denoted as **p < 0.01, ***p < 0.001, ****p < 0.0001, and ns: not significant (*p* > 0.05), analyzed by one‐way ANOVA, followed by Dunnett's multiple comparisons test. Data represent mean ± s.d.

Initially, we used an IVIS system to monitor the distribution of Fe‐MOF/CP in vivo. 4T1 tumor‐bearing mice received intravenous injection of 5 mg k^−1^g Fe‐MOF/CP@ICG and were imaged at 1, 4, 8, 12, and 24 h post‐injection (Figure S43a,b, Supporting Information). The fluorescent intensity at the tumor sites increased over time, reaching a maximum at 12 h postinjection. Fluorescence remained detectable at the tumor sites even at 24 h postinjection, demonstrating the superior tumor‐targeting capability of Fe‐MOF/CP. After 24 h, the mice were sacrificed, and their major organs and tumor tissues were collected for *ex vivo* imaging (Figure S43c,d, Supporting Information). The fluorescence signals are predominantly localized at the tumor sites, confirming the precise accumulation of Fe‐MOF/CP in tumor tissues. Next, the capacities of Fe‐MOF/CP in cholesterol depletion and immune response activation were assessed in a 4T1 tumor‐bearing mouse model (**Figure** **3**a). Filipin staining of tumor slices (Figure 3b,c), revealed strong fluorescence intensities in both Fe‐MOF/P and control groups. However, diminished blue signals were observed in tumor slices of mice receiving ChOx and Fe‐MOF/CP, indicating that ChOx enables Fe‐MOF/P to deplete cholesterol in tumors. As shown in Figure S44 (Supporting Information), Fe‐MOF/CP significantly reduced GPX4 expression in tumor slices, indicating cell ferroptosis. Subsequently, we evaluated in vivo ICD responses by immunohistochemical (IHC) staining of CRT in tumor slices (Figure S45, Supporting Information). As expected, Fe‐MOF/CP elicited robust CRT expression, consistent with the concept that cholesteroldepletion‐boosted ferroptosis can trigger ICD responses (Figure 2l). Furthermore, the antitumor immune responses induced by Fe‐MOF/CP were systematically studied. The transition of tumor‐associated macrophages from M2 to M1 phenotype is essential for antitumor therapy, as M1 macrophages possess strong phagocytosis and antigen presentation abilities. Previous reports indicate that cholesterol accelerates tumor progress by driving tumor‐associated macrophages to an M2 phenotype. Thus, the macrophage phenotype in tumors was characterized by an FCM (Figure S46, Supporting Information). The percentages of M2 macrophages (CD11b^+^F4/80^+^CD206^+^) were 12% for control, 6.4% for ChOx, 4.8% for Fe‐MOF/P, and 2.2% for Fe‐MPF/CP (Figure 3d,e). The decline in M2 by Fe‐MOF/CP validated its significant capacity for macrophage polarization. Moreover, we examined the maturation of DC, another important antigen presentation cell (Figures 3f,g and S47, Supporting Information). Mice receiving ChOx exhibited a slightly higher proportion of DC maturation (10%) (CD11c^+^CD80^+^CD86^+^) compared to the control group (8%). In comparison, mice receiving Fe‐MOF/P and Fe‐MOF/CP showed 17% and 22% maturation, respectively. The enhanced DC maturation benefits T cell priming. As illustrated by FCM analysis (Figures 3h and S48, Supporting Information) and corresponding statistics (Figure 3i), the ratios of cytotoxic T lymphocytes (CD8^+^ cells, gated on CD3^+^) in tumors of Fe‐MOF/CP‐treated mice (15.1%) significantly higher than in the control (3.63%), ChOx (5.67%), and Fe‐MOF/P (10.2%). Afterward, the exhaustion of CD8^+^ T cells was estimated. As shown in Figures 3j,k, and S48 (Supporting Information), the viability (Annexin V^−^) of CD8^+^ cells was 50.3% for control, 16.3% for ChOx, 52.2% for Fe‐MOF/P, and 8.84% for Fe‐MOF/CP. These results indicated that Fe‐MOF/P primed CD8^+^ T cells, but their viability was relatively low. Significantly, Fe‐MOF/CP decreased the ratio of Annexin V^+^CD8^+^ cells compared to Fe‐MOF/P, suggesting that cholesterol depletion by ChOx reverses the exhaustion of CD8^+^ T cells. Additionally, Fe‐MOF/CP elevated the secretion of interferon‐gamma (IFN‐γ) (Figures 3l and S49a, Supporting Information) and tumor necrosis factor α (TNF‐α) (Figures 3m and S49b, Supporting Information) compared to the other three groups, further validating its significant ability to augment antitumor immunity. Histological hematoxylin‐eosin (H&E) staining showed that Fe‐MOF/CP induced damage in tumors (Figure S50, Supporting Information) but had no obvious effects on major organs (Figure S51, Supporting Information), suggesting its tumor therapeutic efficacy and high biosafety. The negligible hemolysis rates of red blood cells after incubating with various concentrations of Fe‐MOF and Fe‐MOF/P, further indicate their good biocompatibility and security (Figure S52, Supporting Information).

### 2.6 Fe‐MOF/CP Inhibits Tumor Metastasis when Combined with PD‐1 Checkpoint Blockade

The functions of primed T cells still are hindered by their surface PD‐1, a common immunosuppressive checkpoint that downregulates the immune system and advances self‐tolerance. Blockading the PD‐1 checkpoint by antibodies enhances antitumor immune responses by intercepting the connections between PD‐1 and tumoral PD‐L1, which has been widely utilized in clinical immunotherapy.^[^ ^49^, ^50^ ^]^ Recent studies have demonstrated that the ICD induced by other therapeutic modalities can be combined with PD‐1 checkpoint blockade for highly efficient tumor treatment and metastasis inhibition.^[^ ^51^ ^]^ Therefore, we investigated the concerted therapeutic efficacy of Fe–MOF/CP and PD–1 antibody (αPD–1) for tumor metastasis inhibition.

**Figure 4:**
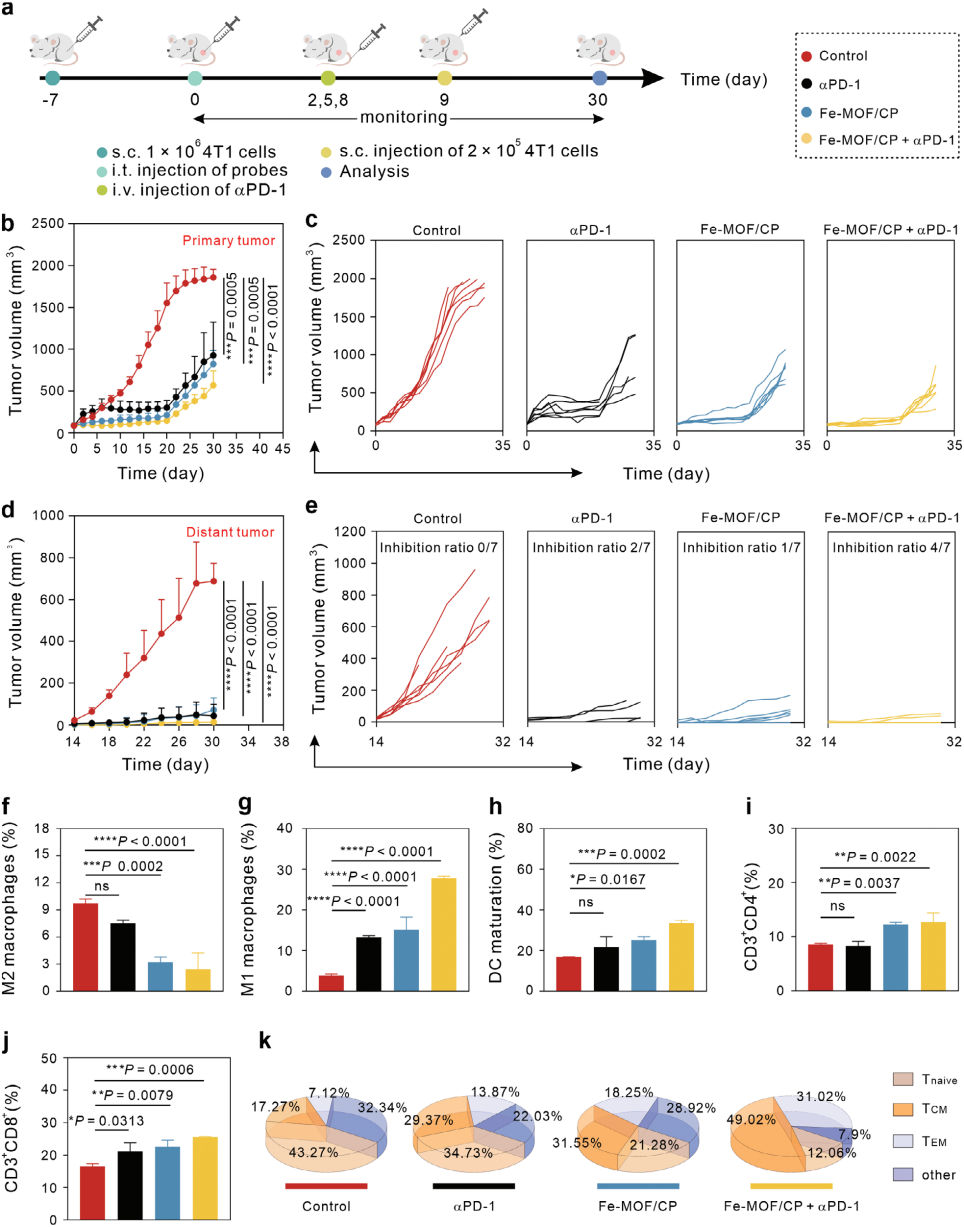
Tumor inhibition by Fe‐MOF/CP combined with αPD‐1 in a bilateral tumor model, *n* = 7. a) Schematic illustration of the treatment procedures. b,c) Total (b) and individual curves (c) depicting primary tumor volumes of mice after various treatments. d,e) Total (d) and individual curves (e) showing distant tumor volumes. f,g) Percentages of M2 macrophages (f) and M1 macrophages (g). h) Statistical analysis of DC maturation. i,j) Statistical outcomes of CD4+ T cells (i) and CD8+ T cells (j). k) Proportion of Tnaive, TCM, and TEM after treatments. Statistical significance denoted as *p < 0.05, **p < 0.01, ***p < 0.001, ****p < 0.0001, and ns: not significant (*p* > 0.05), analyzed by one‐way ANOVA, followed by Dunnett's multiple comparisons test. Data represent mean ± s.d.

We first tested their efficacy in a bilateral tumor model. The treatment scheme is shown in **Figure** **4**a. In this typic experiment, 1 × 10^6^ 4T1 cells were subcutaneously (s. c.) injected into the left leg flanks of mice to form the primary tumor, and Fe‐MOF/CP (200 µg) was injected into tumors 7 days later. On days 2, 5, and 8, mice received with αPD‐1 (1 mg k^−1^g), and 2 × 10^5^ 4T1 cells were subjected to right leg flanks on day 9 to form distant tumors. As shown in Figure 4b,c, tumors in the control group grew rapidly, while both αPD‐1 and Fe‐MOF/P groups delayed the growth of primary tumors. Specifically, Fe‐MOF/CP + αPD‐1 exhibited superior tumor growth retardation compared to other groups. For the distant tumors (Figure 4d,e), the inhibition ratios of 0/7 for the control, 2/7 for αPD‐1, 1/7 for Fe‐MOF/CP, and 5/7 for Fe‐MOF/CP + αPD‐1. Compared to other groups, Fe‐MOF/CP +αPD‐1 significantly inhibited both primary and distant tumors and showed a superior survival rate (Figure S53, Supporting Information) but without obvious changes in body weights (Figure S54, Supporting Information).

Next, we investigated the tumor‐infiltrating immune cells in distant tumors. Figures 4f and S55 and S56 (Supporting Information) illustrate the percentages of M2 macrophages: 9.72% for control, 7.52% for αPD‐1, 3.2% for Fe‐MOF/CP, and 2.42% for Fe‐MOF/CP + αPD‐1. Additionally, the percentages of M1 macrophage were 3.85% for control, 13.2% for αPD‐1, 15.1% for Fe‐MOF/CP, and 27.7% for Fe‐MOF/CP + αPD‐1 (Figures 4g and S55 and S57, Supporting Information). Meanwhile, Fe‐MOF/CP + αPD‐1 exhibited superior ability for DC maturation (33.5%) compared to αPD‐1 (21.7%) and Fe‐MPF/CP (25.03%) (Figures 4h and S58 and S59, Supporting Information). Additionally, mice treated with Fe‐MOF/CP + αPD‐1 showed increased tumor‐infiltrated helper T cells (CD3^+^CD4^+^, Figures 4i and S60, Supporting Information) and CTLs (CD3^+^CD8^+^, Figures 4j and S60, Supporting Information) compared to those in other groups. Notably, when combined with αPD‐1, Fe‐MOF/CP significantly reduced the proportion of naive T cells (T_naive_, CD3^+^CD44^−^CD62L^+^) and elevated the ratios of effective memory T cells (T_EM_, CD3^+^CD44^+^CD62L^−^) and central memory T cells (T_CM_, CD3^+^CD44^+^CD62L^+^, Figures 4k and S60 and S61, Supporting Information). Overall, these findings demonstrate that Fe‐MOF/CP plus αPD‐1 can induce robust antitumor immune responses, showing promise in tumor metastasis and relapse inhibition.

**Figure 5:**
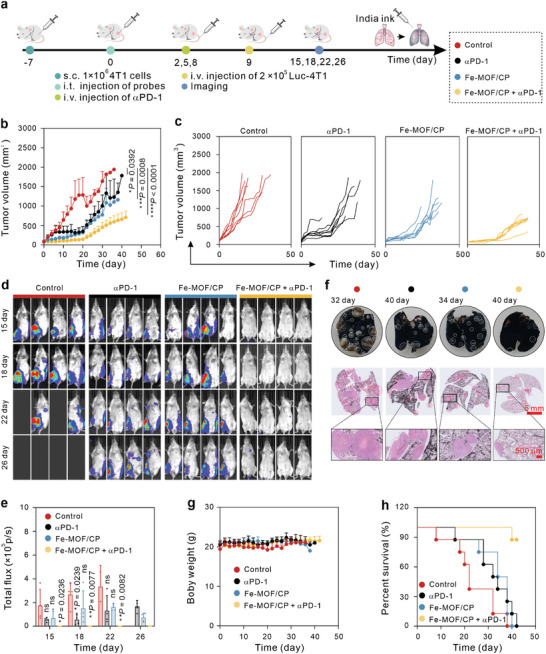
Tumor suppression by Fe‐MOF/CP combined with αPD‐1 in a lung metastasis mouse model. *n* = 8. a) Schematic representation of the treatment processes. b,c) Total (b) and individual tumor volume curves (c) following various treatments. d,e) Representative bioluminescence images (d) and corresponding statistical results (e). f) Representative images of lung tissue stained with H&E and India ink. g,h) Changes in body weights (g) and survival percentage trends (h) of mice throughout the treatment regimen. Statistical significance denoted as *p < 0.05, **p < 0.01, ***p < 0.001, ****p < 0.0001, and ns: not significant (*p* > 0.05), analyzed by one‐way ANOVA, followed by Dunnett's multiple comparisons test. Data represent mean ± s.d.

Subsequently, the synergistic effect of Fe‐MOF/CP with αPD‐1 was further verified in a lung metastasis model, and the treatment process is illustrated in **Figure** **5**a. As shown in Figure 5b,c, Fe‐MOF/CP + αPD‐1 significantly delayed the growth of the primary tumors compared to the other groups. Besides, the metastasis nodules of mice were monitored by an IVIS on days 15, 18, 22, and 26 (Figure 5d), and their relative fluorescence intensities were illustrated in Figure 5e. Groups treated with αPD‐1 and Fe‐MOF/CP displayed fewer bioluminescence signals of metastasis nodules compared to the control group, while the group Fe‐MOF/CP + αPD‐1 showed no obvious signals, implying the superb metastasis inhibition efficacy of this combination. Additionally, the metastatic nodules in lung tissues were detected by India ink perfusion and H&E staining (Figure 5f). Unsurprisingly, both the Fe‐MOF/CP and αPD‐1 groups exhibited a certain degree of canceration and fewer metastatic nodules compared to the control group, while lungs from Fe‐MOF/CP + αPD‐1 group showed less canceration and metastatic nodules than other groups. Specifically, Fe‐MOF/CP + αPD‐1 had no obvious effects on body weights (Figure 5g) but significantly prolonged mouse survival (Figure 5h).

**Figure 6:**
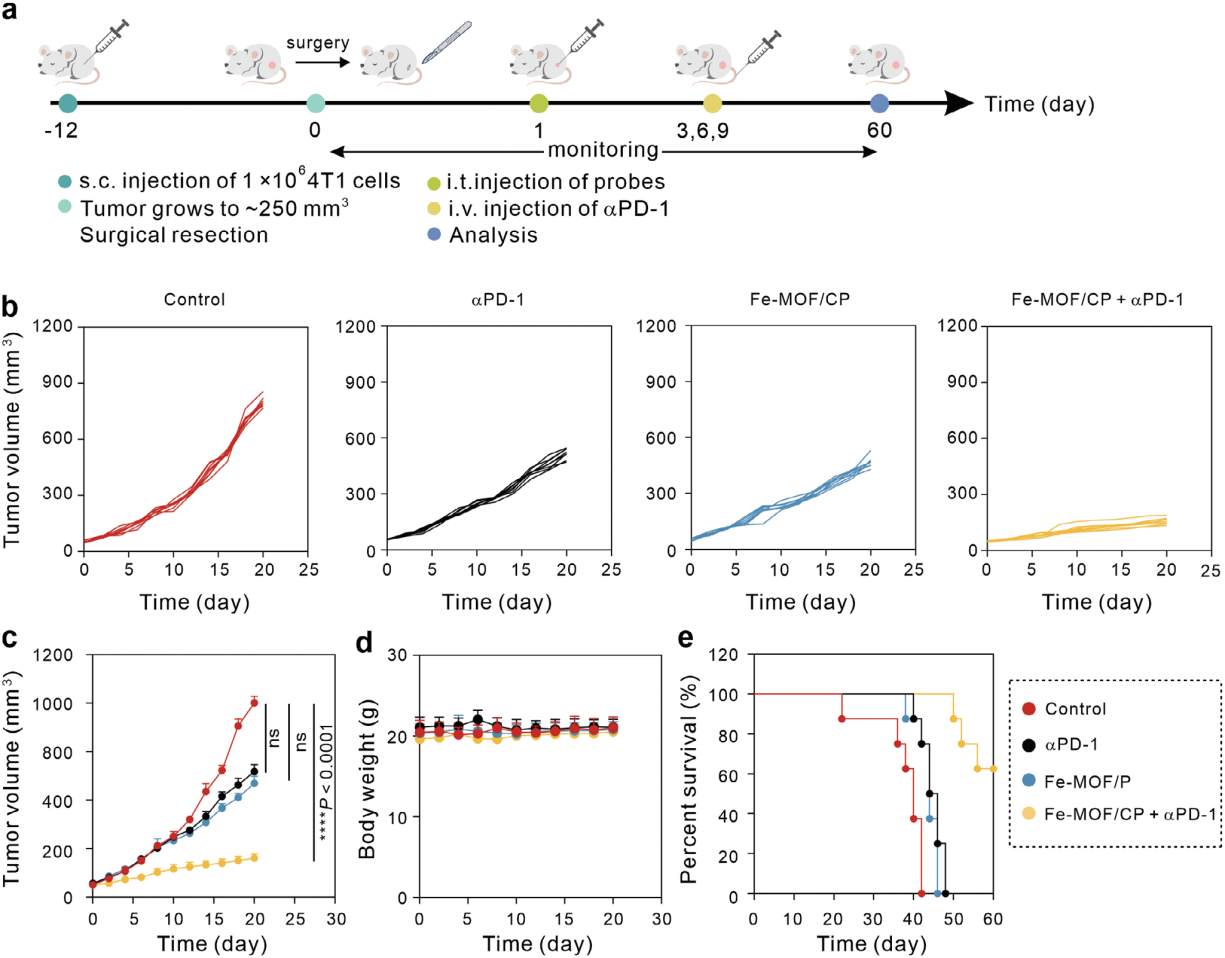
Fe‐MOF/CP inhibits locoregional cancer recurrence when combined with αPD‐1, *n* = 8. a) Schematic illustration of treatment processes. b,c) The individual (b) and total curves (c) of tumor volumes of mice after various treatments. d,e) The body weights (d) and survival curves (e) of mice during treatments. Statistical significance is denoted as ****p < 0.0001 and ns: not significant (*p* > 0.05), analyzed by one‐way ANOVA, followed by Dunnett's multiple comparisons test. Data represent mean ± s.d.

Finally, the locoregional tumor recurrence mouse model was established to evaluate the synergistic effect of Fe‐MOF/CP and αPD‐1, and the treatment process is illustrated in **Figure** **6** **a**. In the tipical experiment, 1 × 10^6^ 4T1 cells were s. c. injected into the left leg flanks of mice, and tumors were surgically resected to ≈50 mm^3^ when their volumes reached ≈250 mm^3^. Then, mice were administrated with assigned formulations. As displayed in Figure 6b,c, tumors of mice in the control group grew rapidly, while both αPD‐1 and Fe‐MOF/CP delayed the tumor recurrence. Significantly, Fe‐MOF/CP + αPD‐1 obviously inhibited the growth of tumors. The negligible body weight changes (Figure 6d) and prolonged mouse survival (Figure 6e) further revealed the notable biosafety and synergistic therapeutic efficacy of the Fe‐MOF/CP and PD‐1 checkpoint blockade, respectively.

## 3 Conclusion

In our study, we introduce Fe‐MOF/CP, a hybrid nanozyme based on Fe‐MOF, engineered for tumor ferroptosis and immunotherapy by strategically incorporating ChOx into Fe‐MOF nanoparticles. Fe‐MOF/CP retains the POD and GSHox‐mimicking activities of Fe‐MOF, which induce cellular ferroptosis by generating tumor‐specific ROS and depleting GSH. The incorporation of ChOx into Fe‐MOF/CP hybrid nanozyme facilitates cholesterol depletion and increases H_2_O_2_ levels within tumors, disrupting cellular lipid rafts and leading to decreased expression of anti‐ferroptosis proteins GPX4 and FSP1, thus promoting ferroptosis. Moreover, ChOx enhances ICD responses and reduces PD‐L1 expression in Fe‐MOF‐treated cancer cells. In vivo experiments demonstrated that cholesterol depletion augments the antitumor immune responses elicited by Fe‐MOF, including DC maturation, macrophage polarization, priming of CD8^+^ T cells, and reversion of CD8^+^ T cell exhaustion. When combined with PD‐1 checkpoint blockade, Fe‐MOF/CP synergistically enhances antitumor immune responses and activates memory T cells. Notably, Fe‐MOF/CP exhibits significant therapeutic efficacy in bilateral, metastasis, and recurrent mouse models. This study highlights the synergistic therapeutic potential of combining ferroptosis and cholesterol depletion in tumor treatment. Our work highlights the potential of Fe‐MOF/CP as a multifunctional therapeutic agent that integrates ferroptosis and immunotherapy for cancer treatment. By amplifying ROS levels and depleting cholesterol, this nanozyme addresses the intrinsic resistance mechanisms of cancer cells, paving the way for more effective and durable cancer therapies. Future studies will focus on optimizing the delivery and targeting of Fe‐MOF/CP to further enhance its therapeutic efficacy and minimize potential side effects. This research sets a foundation for the development of advanced nanozyme‐based treatments that can synergize ferroptosis and immune responses to combat cancer.

## Conflict of Interest

The authors declare no conflict of interest.

## Supporting information

Supporting Information

## Data Availability

The data that support the findings of this study are available from the corresponding author upon reasonable request.
