# Supplementary material for: Cholesterol Depletion‐Enhanced Ferroptosis and Immunotherapy via Engineered Nanozyme
Source: Adv Sci (Weinh). 2024 Aug 9;11(38):2405826. doi: 10.1002/advs.202405826 (PMC11481222; doi:10.1002/advs.202405826)
Supplement: Supplementary file 1 — Supporting Information [file ADVS-11-2405826-s001.docx]

Supporting Information

**Cholesterol Depletion-Enhanced Ferroptosis and Immunotherapy *via* engineered Nanozyme**

Tingjie Bai^†^, Panpan Xue^†^, Sijie Shao, Shuangqian Yan^*^, and Xuemei Zeng^*^

T. Bai, Prof. X. Zeng

Key Laboratory of Microbial Pathogenesis and Interventions of Fujian Province University, Biomedical Research Center of South China, College of Life Sciences,

Fujian Normal University

Fuzhou 350117, China.

E-mail: [xmzeng@fjnu.edu.cn](mailto:xmzeng@fjnu.edu.cn)

P. Xue, S. Shao, Prof. S. Yan

The Straits Institute of Flexible Electronics (SIFE, Future Technologies), Straits Laboratory of Flexible Electronics (SLoFE)

Fujian Normal University

Fuzhou 350117, China

E-mail: [ifeshqyan@fjnu.edu.cn](mailto:ifeshqyan@fjnu.edu.cn)

**†** T. Bai and P. Xue contributed equally.

**Experimental Produres**

**Materials and methods**

**Materials**: Pluronic® F-127 and indocyanine green (ICG) were bought from Sigma-Aldrich. Iron (III) chloride (FeCl_3_), 2-aminoterephthalic acid, cholesterol oxidase (ChOx), o-phenylenediamine (OPD), methylene blue (MB), sodium chloride (NaCl), and Triton X-100 were purchased from Aladdin. Acetate was got form Sinopharm. DSPE-mPEG (2000) was sourced from Shanghai Yare Co., LTD. Hydrogen peroxide (H_2_O_2_) and formaldehyde were acquired from Xihua Co., LTD. CCK-8, Lipid peroxidation (LPOs) kit was obtained from Dojindo Co., [Ru(dpp)_3_Cl_2_] was received from Macklin. Amplex Red, 4′,6-diamidino-2-phenylindole (DAPI), RIPA lysis buffer, protease and phosphatase inhibitor cocktail (50X), BCA assay kit, SDS-PAGE loading buffer, anti-GPX4 (Cat# AF7020), and ATP assay kit were acquired from Beyotime. ^•^OH fluorescent detection kit was obtained from Bestbio. Filipin complex was got from MedChemExpress. Anti-FPS1 (Cat# 20886-1-AP), HRP-conjugated affinipure goat anti-mouse IgG (H+L) (Cat# 15014), HRP-conjugated affinipure goat anti-Rabbit IgG(H+L) (Cat# 15015), and anti-calreticulin polyclonal (CRT) (Cat# 10292-1-AP) were gained from Proteintech. Anti-α-Tubblin was acquired from Cell Signaling Technology. Anti-4-HNE was bought from Origo. TMB solution, skim milk powder, Tween-20, Tris (hydroxymethyl) aminomethane, sodium dodecylsulfate, glycine, and crystal violet were obtained from Solarbio. 10% PAGE gel fast preparation kit was purchased from Epizyme. 0.45 µm immobilon- PVDF was got from Millipore. SuperKine™ west femto maximum sensitivity substrate was gained from Abbkine. Matrigel® matrix and collagen typeⅠ were got from Corning. Goat anti-rabbit IgG(H+L) (AF594 conjugated) (Cat# E-AB-1060) was acquired from Elabscience. HMGB1 ELISA kit was purchased from Mlbio. TNF-α and IFN-γ ELISA kits were got from Jingmei Biotechnology. Co., IL-4 protein and GM-CSF/CSF2 protein were purchased from Sinobiological. Anti-mouse PD-1 (Cat# BE0273-100MG) was acquired from Univ Company. Transcription factor buffer set was got from BD Pharmingen. DMEM, phosphate buffer solution (PBS), and fetal bovine serum (FBS) were bought from Gibco. CTLL-2 cell culture medium was obtained from HaiXing bio. All antibodies used in flow cytometry are listed in Table S2.

**Instruments:** TEM images were obtained using a transmission electron microscope (Hitachi, HT7700). X-Ray diffraction patterns were acquired with a Rigaku Miniflex600 X-ray diffractometer. BET results were recorded using ASAP2020 Version 4.03. software from Micromeritics. XPS spectra were obtained using a Thermo SCIENTIFIC Nexsa X-ray photoelectron spectrometer. Zeta potential and hydrodynamic size were measured using Zetasizer Nano ZS (Malvern). FTIR spectra were surveyed using a Thermo Nicolet IS 50 Fourier transform infrared spectrometer.

**Synthetic procedures of nanopaticles**

For the synthesis of Fe-MOF, 0.64 g Pluronic® F-127 dissolved in 50 mL water was mixed with 0.714 g of FeCl_3_ dissolved in 10 mL of water. The resulting mixture was transferred into a 50 mL conical bottle and subjected to ultrasonication for 10 min followed by stirring for 1.5 h. Subsequently, 0.6 mL of glacial acetic acid was added and the mixture was stirred for another 1.5 h. Then, 240 mg of 2-amino-terephthalic acid was added and stirred for another 2 h. The mixture was then transferred to a high-temperature reactor to react for 24 h at 110 ℃. Finally, Fe-MOF was obtained by centrifugation (8000 rpm, 10 min) for several times.

For the synthesis of Fe-MOF/CP, 4 mg of Fe-MOF and 3 mg of ChOx were dissolved in 1 mL of water and stirred overnight. Then, 4 mg of DSPE-mPEG was added, and the mixture was stirred for another 6 h. Finally, the mixture was centrifuged (8000 rpm, 10 min) several times to obtain Fe-MOF/CP.

For the synthesize Fe-MOF/CP@ICG, 1 mg each of Fe-MOF/CP and ICG were dissolved in 1 mL of water and stirred overnight. The resulting mixture was subsequently centrifuged multiple times (8000 rpm, 10 min) to yield Fe-MOF/CP@ICG.

The release of Fe^2+^ and ChOx from Fe-MOF/CP was determined by incubating Fe-MOF/CP in pH 7.4 or pH 6.5 buffer at 37 ◦C for various time intervals. The concentration of Fe^2+^ was determined using o-phenanthroline, and the concentration of ChOx was measured using BCA protein assay kit.

**Cholesterol depletion ability of Fe-MOF/CP**

PBS, 60 μg/mL ChOx, 100 μg/mL Fe-MOF/P, and Fe-MOF/CP were mixed with 4 μM cholesterol at 37 ◦C for 24 h, and cholesterol content measured using a cholesterol assay kit.

**Catalytic stability of Fe-MOF/CP**

Fe-MOF/CP (1 mg/mL) and ChOx (60 μg/mL) were incubated at different temperatures (25, 37, 50, 60, and 70 °C) for 2 h and pH levels (4, 5, 6, 7, 8, and 9) for 48 h. Subsequently, 60 μg/mL cholesterol was added to the mixtures and incubated for an additional hour. Enzyme activity was assessed using a cholesterol content kit. The enzyme activity of Fe-MOF/CP and ChOx at 45 °C and pH = 5 was equally defined as 100%.

**^•^OH generation ability of Fe-MOF**

For evaluate ^•^OH generation ability of Fe-MOF, an EPR spectrometer (Bruker-EMXPlus-10) was used to determine the ^•^OH generation. 250 μg/mL Fe-MOF incubated with 10 mM H_2_O_2_ for 15 min and added 100 mM DMPO to trap the ^•^OH. Besides, TMB, MB, and OPD could react with ^•^OH to generate colore variation. 250μg/mL Fe-MOF mixed with or without 10 mM H_2_O_2_ for 30 min, and then the absorbance at 650 nm, 617 nm, and 450 nm was measured respectively using a microplate reader (Model 1510, Thermo Fish). Subsequently, the absorption peak of 200-1000 nm was detected using an ultraviolet absorption spectrometer (UV-8000S, Shanghai Metash Instruments Co., Ltd).

For evaluate ^•^OH generation ability of Fe-MOF across various pH levels, 250 μg/mL Fe-MOF was incubated with or without 10 mM H_2_O_2_ for 10 min, and then the absorbance at 650 nm was quantified *via* a microplate reader.

For the investigation of POD-like catalytic activity, 250 μg/mL of Fe-MOF was reacted with TMB in the present of varying concentrations of H_2_O_2_ (5, 10, 15, 30, and 50 mM), and the absorbance intensity at 650 nm was monotored using a microplate reader for 30 min.

**GSH depletion ability of Fe-MOF**

Fe-MOF at a concentration of 100 μg/mL was mixed with 10 mM GSH and stirred for 12 h. The remaining GSH in the supernatant was then reacted with DTNB, and the absorption intensity at 410 nm was measured.

For GSHox-like catalytic activity, 250 μg/mL of Fe-MOF was incubated with various concentrations of GSH (1, 3.75, 6.25, 12.5, and 25 mM). The supernatant was then reacted with DTNB until the reaction was complete. The absorbance intensity at 430 nm was measured over a period of 30 min.

**Cell culture**

Murine breast cancer 4T1 cells and luciferase-expressed 4T1 (Luc-4T1) cells were cultured in DMEM media supplemented with 10% FBS and 1% penicillin-streptomycin. CTLL-2 cells were cultured with 1640 media supplemented with 10% FBS, 100 U/mL IL-2, 1 μg/mL Con A, and 1% penicillin-streptomycin. All cells were maintained in a cell incubator at 37 °C under 5% CO_2_.

**Intracellular** **cholesterol depletion**

For fluorescent images, 4T1 cells inoculated in confocal dishes and treated with 60 μg/mL ChOx, 100 μg/mL Fe-MOF/P, and Fe-MOF/CP for 24 h. Afterward, 50 μg/mL Filipin complex was added to the different groups for 2 h, and images were captured using a LSCM (Zeiss, LSM780).

For cholesterol content assays, 4T1 cells were subjected to detection using a cholesterol measurement kit under various treatments.

**Intracellular GSH consumption evaluation**

4T1 cells were seeded in six-well plates and treated with 60 μg/mL ChOx, 100 μg/mL Fe-MOF/P, and Fe-MOF/CP for 24 h. Cellular GSH content was measured using a commercial GSH assay kit. Additionally, 4T1 cells were cultured overnight in small confocal dished and cultured with 60 μg/mL ChOx, 100 μg/mL Fe-MOF/P, and Fe-MOF/CP for 24 h. Cell were then stained with 100 μM Bromodiamine (a GSH detection probe) at 37 ℃ for 30 min and imaged using CLSM.

**Intracellular Fe^2+^ evaluation**

4T1 cells were seeded in four small confocal dishes and cultured with 60 μg/mL ChOx, 100 μg/mL Fe-MOF/P, and Fe-MOF/CP for 24 h. The cells were then incubated with 1 μM FerroOrange (a ferric ion detection probe) at 37 ℃ for 30 min. Finally, the cell samples were fixed with 4% paraformaldehyde for 15 min, stained with DAPI for 10 min, and imaged using a CLSM.

**Intracellular O_2_ evaluation**

4T1 cells were seeded in small confocal dishes and cultured with 60 μg/mL ChOx, 100 μg/mL Fe-MOF/P, and Fe-MOF/CP for 24 h. Then, the cells samples were incubated with 50 μM [Ru(ddp)_3_Cl_2_] at 37 ℃ for 2 h. Finally, cell samples were fixed with 4% paraformaldehyde for 15 min, stained with DAPI for 10 min, and imaged using a CLSM.

**Intracellular H_2_O_2_ evaluation**

4T1 cells were seeded in small confocal dishes and cultured with 60 μg/mL ChOx, 100 μg/mL Fe-MOF/P, and Fe-MOF/CP for 24 h. Then, the cell samples were treated with 500 μL serum free DMEM medium supplemented with HRP (3 U) and Amplex red (2 μL) and incubated at 37 ℃ for 30 min before imaging in a CLSM.

**Intracellular ^•^OH evaluation**

4T1 cells were seeded in small confocal dishes and cultured with 60 μg/mL ChOx, 100 μg/mL Fe-MOF/P, and Fe-MOF/CP for 24 h. Then, the cell samples were treated with 1 mL serum free DMEM medium and 1μL ^•^OH fluorescent detection solution, followed by incubation at 37 ℃ for 30 min. After washing with PBS twice, images of cell samples were captured using a CLSM.

**Fluidity membrane detection**

After being treated with 60 μg/mL ChOx, 100 μg/mL Fe-MOF/P, and Fe-MOF/CP for 24 h, 4T1 cells were trypsinized and stained with 1-pyrenedodecanoic acid for 5 min in dark. Subsequently, using a fluorescence spectrophotometer (F-7100, Hitachi), the fluorescence spectra were scanned with an excitation wavelength of 380 nm and an emission wavelength of 380-580 nm. The fluidity of each group was assessed by comparing according the ratio of excimer (475 nm) to pyrene monomer (397 nm).

**Intracellular LPOs evaluation**

4T1 cells were seeded in small confocal dishes and cultured with 60 μg/mL ChOx, 100 μg/mL Fe-MOF/P, or Fe-MOF/CP for 24 h. The cells were then stained with 6 μM Liperfluo at 37 ℃ for 30 min and imaged using a CLSM. In addition, 4T1 cells were seeded in a six-well plate overnight and subsequently cultured with the same concentrations of ChOx, Fe-MOF/P, or Fe-MOF/CP for 24 h. Following this, the cells were stained with 6 μM Liperfluo at 37 ℃ for 30 min and collected for flow cytometry analysis.

**Western blot assay**

4T1 cells were inoculated a six-well plate (2 × 10^6^ cells per well) and incubated with 60 μg/mL ChOx, 100 μg/mL Fe-MOF/P, and 100 μg/mL Fe-MOF/CP. The cells were then homogenized in RIPA cell lysis buffer, and protein concentration was measured by a BCA kit. Equal amounts of protein were loaded into SDS-PAGE loading buffer, separated by electrophoresis, and transferred to PVDF membranes. The membranes were blocked with skim milk and incubated overnight at 4 ℃ with anti-4-HNE (1: 500) anti-GPX4 (1: 1000), anti-FPS1 (1: 1000), anti-CD59 (1: 1000), and anti-PD-L1 (1: 1000). After washing with TBST solution, the membranes were incubated with secondary antibodies against rabbit IgG (1: 5000) or mouse IgG (1: 5000). Expression levels were normalized for minor differences in loading.

**Cell viability and apoptosis analysis**

For the cell viability assay, 4T1 cells were evenly seeded into a 96-well plate (5 × 10^4^ cells per well) and incubated for 12 h. Various concentrations of Fe-MOF/P and Fe-MOF/CP (0, 50, 100, 150, 200, and 300 μg/mL, representing the Fe-MOF/P concentration) were added and incubated for 24 h. Cell viability was assessed using a CCK-8 kit.

For cell apoptosis assay, 4T1 cells were grown into a six-well plate overnight (5 × 10^5^ cells per well). PBS, 60 μg/mL of ChOx, 100 μg/mL of FeMOF/P, 100 μg/mL of FeMOF/CP were added and incubated for 24 h. Cell samples were washed with ice-cold PBS, stained with PI for 15 min in the dark, and the 400 μL Binding Solution was added before FCM analysis (Agilent, Novoexpress).

For the T cells apoptosis assay, CTLL2 cells were seeded overnight in six-well plates at a density of 5 × 10^5^ cells per well. PBS, 60 μg/mL ChOx, 100 μg/mL FeMOF/P, and 100 μg/mL FeMOF/CP were added and incubated for 24 h. Cell samples were washed with ice-cold PBS, stained with Annexin V-FITC/PI for 15 min in the dark, and incubated with 400 μL Binding Solution before FCM analysis.

**Migration assay**

1 × 10^4^ 4T1 cells were resuspended in 200 μL serum-free DMEM and added to each upper transwell chamber. Then, transwells were placed on top of a 24 well plate containing 500 µL complete DMEM media (10% FBS and 1% streptomycin and penicillin) and incubated with 60 μg/mL ChOx, 100 μg/mL FeMOF/P, and 100 μg/mL FeMOF/CP for 24 h. Cells were fixed with 4% paraformaldehyde for 20 min and stained with crystal violet for 10 min. Finally, cell samples were washed with PBS and gently wiped the cells in the inner wall with a cotton swab before being photographed with a microscope.

**Adhesion assay**

Fibronection (Collagen TypeⅠ) was diluted to 10 μg/mL with pre-cooled serum-free DMEM and 50 μL was added to each well of 96 well plates at 4 ℃ overnight. 5 × 10^4^ cells were resuspended in 200 μL serum-free DMEM and added to the each well. Cells were cultured with 60 μg/mL ChOx, 100 μg/mL FeMOF/P, and 100 μg/mL FeMOF/CP for 24 h, followed by the removal of unattached cells. Subsequently, attached cells were fixed with 4% paraformaldehyde for 20 min and stained with crystal violet for 10 min. Finally, adherent cells were observed and imaged using a microscope.

**Invasion assay**

Matrigel^®^ Matrix was diluted to 5 mg/mL with serum-free DMEM and 50 μL was added to each transwell chamber at 37 ℃ for 1 h. 1 × 10^4^ cells were resuspended with 200 μL serum-free DMEM and added to the each upper transwell chamber. The transwells were then placed on top of a 24-well plate containing 500 µL complete DMEM media and incubated with 60 μg/mL ChOx, 100 μg/mL FeMOF/P, and 100 μg/mL FeMOF/CP for 24 h. Cells were fixed with 4% paraformaldehyde for 20 min and stained with crystal violet for 10 min. Finally, the cells were washed with PBS, and those on the inner wall were gently wiped with a cotton swab before photographing with a microscope.

**Wound healing assay**

4T1 cells were seeded in 24-well plates (5 × 10^5^ cells per well) and cultured cells until they almost covered the bottom of the plates. A scratch was then simulated in the cell samples, and images were captured with a microscope, marking the starting point (0 h). Subsequently, cells were incubated with 60 μg/mL ChOx, 100 μg/mL FeMOF/P, and 100 μg/mL FeMOF/CP for 24 h, and images were captured again at equal intervals.

***In vitro* ICD effect evaluation**

4T1 cells were seeded in small confocal dishes and incubated overnight. The cells were then treated with 60 μg/mL ChOx, 100 μg/mL FeMOF/P, and 100 μg/mL FeMOF/CP for 24 h. The supernatant was collected to measure ATP release. For CRT exposure, cell samples were fixed with 4% paraformaldehyde for 30 min, followed by incubation with CRT primary antibody (1: 200)at 4℃ for 30 min and secondary antibody goat anti-rabbit IgG (H+L) (AF594 conjugated) (1: 1000) at 4℃ for 1 h. To assess HMGB1 secretion, cell samples were stained with a primary HMGB1 antibody (1: 200) overnight and a CoraLite488-conjuated Goat anti-rabbit lgG (H+L) (1: 1000) for 1 h after fixation with 4% paraformaldehyde for 30 min. Finally, cells were stained with DAPI and imaged using a CLSM.

***In vivo* biodistribution**

All the mouse experiments adhered to protocols approved by the Animal Experimental Ethics Committee of the Fujian Normal University (Approval No. IACUC-20220006). Female Balb/c mice (6 weeks) were obtained from Shanghai Slack Laboratory Animal Co., Ltd.. 1 × 10^6^ 4T1 cells were injected subcutaneously in to the mice. When the tumor volume reached 200 mm^3^, the tumor-bearing mice were i.v. injected with 5 mg/kg Fe-MOF/CP@ICG (n = 3). After that, the mice were imaged using an IVIS system at 1, 4, 8, 12, and 24 h post-injection. At 24 h post-injection, the mice were sacrificed, and their major organs and tumor tissues were colloected for *ex vivo* imaging.

***In vivo* immune response evaluation**

Female Balb/c mice (6 weeks) were obtained from Shanghai Slack Laboratory Animal Co., Ltd.. Female Balb/c mice were injected with 1 × 10^6^ 4T1 under their skin. When the tumor volume reached 100 mm^3^, tumor-bearing mice were divided into 4 groups (n = 5) to intratumor injection with various probes (PBS, 120 μg ChOx, 200 μg Fe-MOF/P, and 200 μg [Fe-MOF/CP, representing the Fe-MOF concentration). After](mailto:Fe-MOF/C@P).After) 14 days of treatments, tumors were examined by H&E staining, Filipin complex staining, and IHC staining of CRT and GPX4. Major organs (heart, liver, spleen, lung, and kidney) were also fixed, sectioned, and subjected to H&E staining. Additionally, tumors and lymph nodes were collected and prepared into single-cell suspensions. To analyze macrophage polarization, anti-mouse CD11b-APC/Cy7, anti-mouse F4/80-PE/CF594, and anti-mouse CD206-AF647 were stained with tumor cells. Cells were stained with anti-mouse CD8a-PERCY5.5, anti-mouse CD3-PE/Cy7, and Annexin V-FITC to analyze T cell exhaustion. Anti-mouse CD86-PE, anti-mouse CD80-FITC, and anti-mouse CD11c-BV421 were stained with cells from lymph nodes to analyze DC maturation. Serum samples were obtained, and the concentration of TNF-α and IFN-γ were measured using ELISA kits following the manufacturer’s instructions.

**Hemolysis assay**

Blood samples were collected from mice’s eyes and centrifuged at 3000 rpm for 5 min to isolate red blood cells. The cells were then washed with PBS and diluted to 2% concentration. Subsequently, the cells were incubated with different concentrations of Fe-MOF and FeMOF/P for 1 h at 37 ℃. Equal volume of PBS and 0.5% Triton X-100 were added as negative (N) and positive controls (P), respectively. Hemolysis rates were determined by the absorbance of the mixtures at 576 nm using the formula:

Hemolysis (%) = (OD576_sample_ - OD576_N_) / (OD576_P_ - OD576_N_) × 100%

***In vivo* anti-****abscopal activity**

To establish a bilateral model, 4T1 cells (1 × 10^6^ cells) were injected into the left leg flank subcutaneous tissues of Balb/c mice to simulate primary tumors. Once the tumor volume reached 100 mm^3^, the tumor-bearing mice were randomly divided into four groups (7 mice for each group) and subsequently received i.t. injection of different agents (120 μg of ChOx, 200 μg of Fe-MOF/P, and 200 μg of [Fe-MOF/CP, representing the Fe-MOF concentration).](mailto:Fe-MOF/C@P).After) Additionally, the mice were i.v. treated with αPD-1 (1 mg/kg) on days 2, 5, and 8. On day 9, 2 × 10^5^ 4T1 cells were injected into the right leg flank subcutaneous tissues of Balb/c mice to mimic distant tumors. Mice were sacrificed once the tumors reached 2000 mm^3^.

The tumor, lymph, and spleen tissues were extracted and processed into single-cell suspensions for immunoassays. DC maturation in lymph was assessed using anti-mouse CD11c-BV421, anti-mouse CD80-FITC, and anti-mouse CD86-PE. Additionally, macrophage polarization was evaluated using anti-mouse CD11b-APC/Cy7, anti-mouse F4/80-PE/CF594, anti-mouse CD86-APC, and anti-mouse CD206-FITC. Intratumoral immune cells were analyzed for CD4^+^ helper T cells and CD8^+^ cytotoxic T cells using anti-mouse CD3-PE/Cy7, anti-mouse CD4-BV421, and anti-mouse CD8-APC/Cy7. Memory T cells from the spleen were stained with anti-mouse CD3-APC, anti-mouse CD44-FITC, and anti-mouse CD62L-BV650.

***In vivo*** **antimetastatic activity**

Female Balb/c mice were inoculated with 4T1 cells (1 × 10^6^ cells) in the left leg flank. Once the tumor volume reached 100 mm^3^ (n = 8), the mice were i.t. injected with [Fe-MOF/CP.](mailto:Fe-MOF/C@P).After)  two days later, the mice were i.v. administered αPD-1 (1 mg/kg) on days 2, 5, and 8. On day 9, 2 × 10^5^ Luc-4T1 cells were i.v. injected into mice. Throughout the experiment, the body weight and survival of tumor-bearing mice were closely monitored, and euthanasia was performed when the tumor volume exceeded 2000 mm^3^. Upon euthanasia, the lungs of decreased mice were dissected, filled with Indian ink solution, and washed for the counting of metastatic foci. Additionally, bioluminescence imaging was conducted on Balb/c mice by i.p. injection of D-luciferin potassium salt (150 mg/kg) on days 15, 18, 22, and 26, followed by imaging with IVIS (PerkinElmer) after a 10-min interval.

***In vivo* anti-recurrent activity**

In the recurrent mode, Balb/c mice were initially injected with 1 × 10^6^ 4T1 cells into the left leg flank on day 0. Once the tumor volume reached 250 mm^3^, tumors were surgically excised to a size of approximately 50 mm^3^. Subsequently, the mice were treated with different formulations: control, αPD-1, Fe-MOF/CP, and Fe-MOF/CP + αPD-1. αPD-1 was i.v. injected on days 3, 6, and 9 (n=8). Tumor volume was measured every other day over a 60-day period.

**Statistical analysis**

All statistical analyses were performed using GraphPad Prism software. Results are presented as mean ± SD. Statistical significance was determined using one-way ANOVA analysis calculated Statistical significance. Significance levels were denoted as follows: *P < 0.05; **P < 0.01; ***P < 0.001; ****P < 0.0001; ns indicates not significant (P > 0.05).

**Supplementary Figures and Tables**


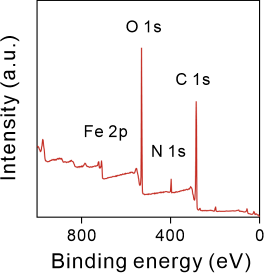


**Figure S1**. XPS spectra of Fe-MOF.


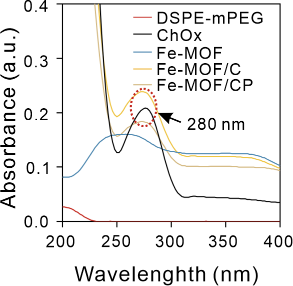


**Figure S2**. UV-vis spectra of DSPE-mPEG, ChOx, Fe-MOF, Fe-MOF/C, and Fe-MOF/CP. Red dotted circle indicates the absorbance of ChOx at 280 nm.


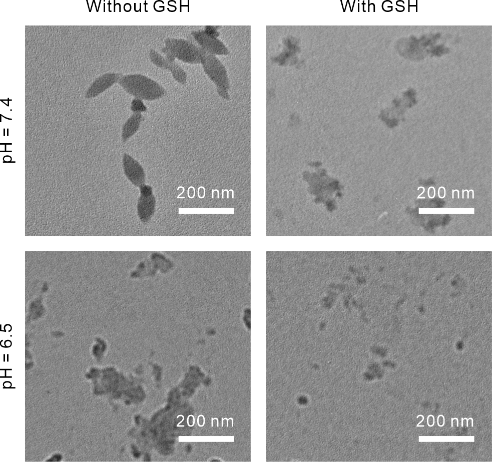


**Figure S3**. TEM images of Fe-MOF with different treatments for 12 h. GSH: 10 mM. Scale bars: 200 nm.


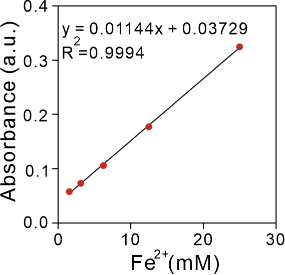


**Figure S4**. Fe^2+^ standard curve profiled by absorption of o-Phenanthroline at 510 nm.


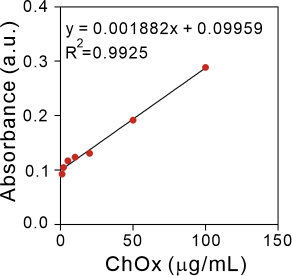


**Figure S5**. ChOx standard curve obtained by a BCA protein assay kit.


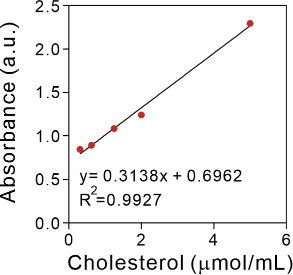


**Figure S6**. Cholesterol standard curve obtained using a cholesterol kit.


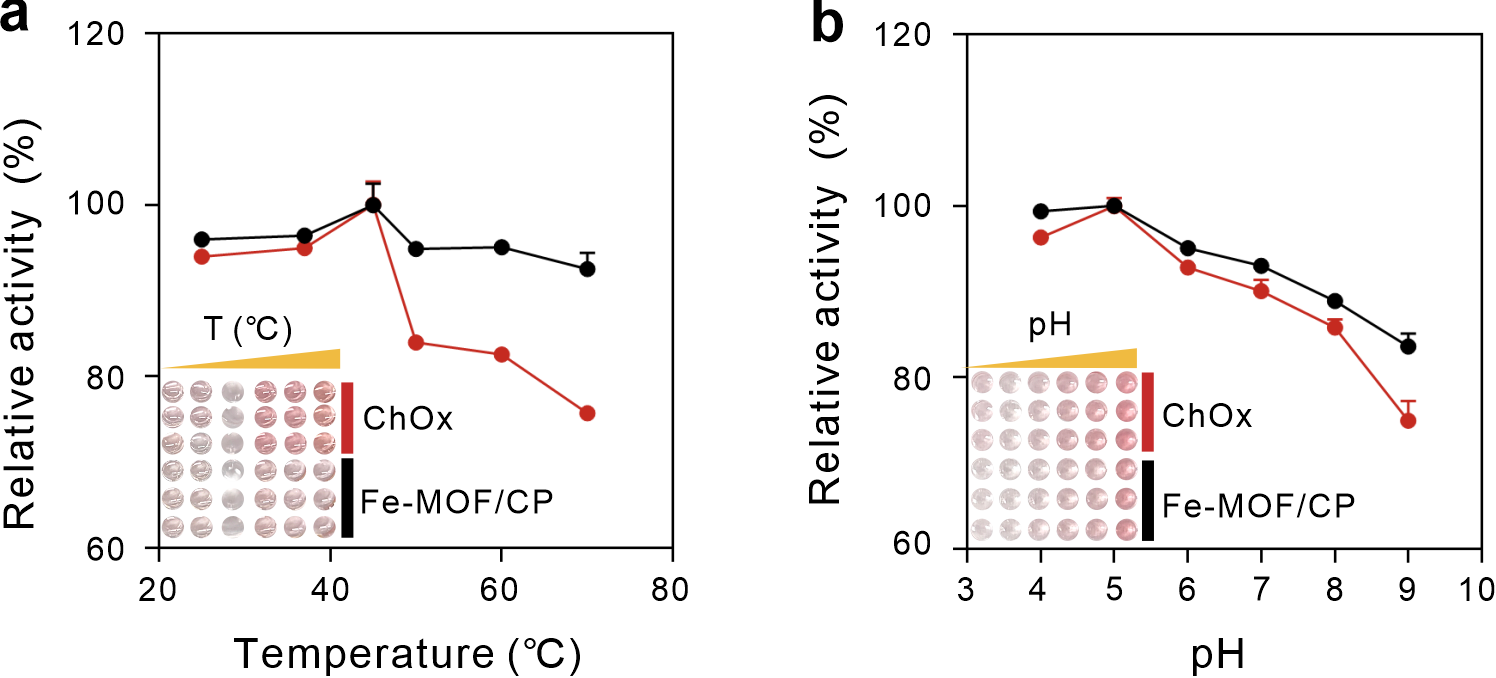


**Figure S7.** (a, b) The relative activity of temperature (a) and pH (b) for ChOx and Fe-MOF/CP.


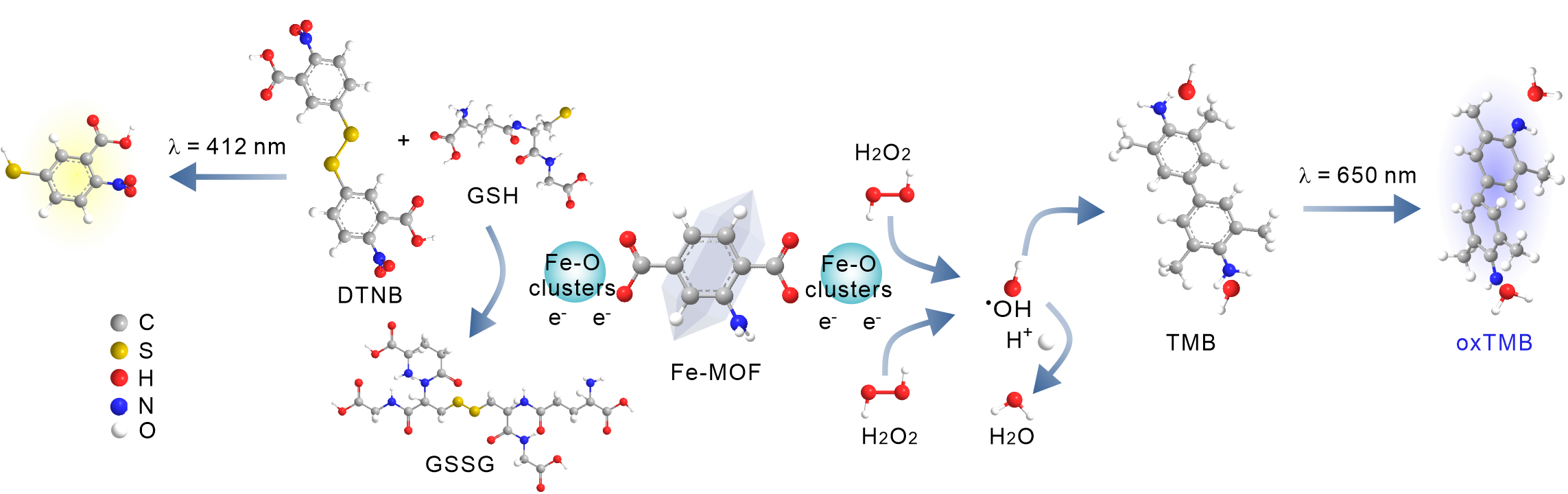


**Figure S8**. The schematic illustrated the POD-like and GSHox-like enzyme of Fe-MOF.


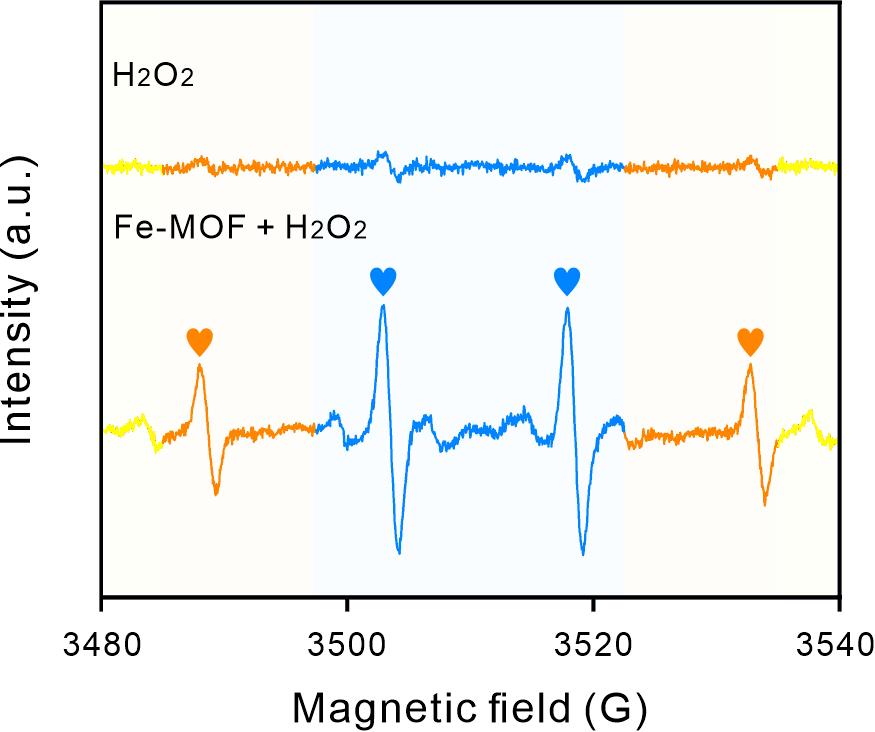


**Figure S9**. ^•^OH radical detection using EPR spectroscopy.


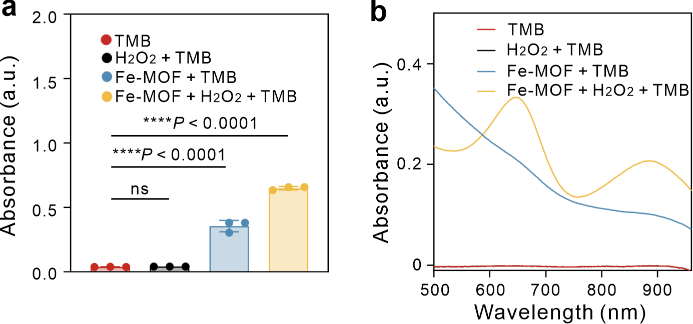


**Figure S10.** (a, b) Absorption intensity of oxTMB at 650 nm (a) (n = 3) and UV-vis absorbance spectra (b). Statistical significance denoted as ****P < 0.0001 and ns: not significant (P > 0.05), analyzed by one-way ANOVA, followed by Dunnett’s multiple comparisons test. Data represent mean ± s.d.


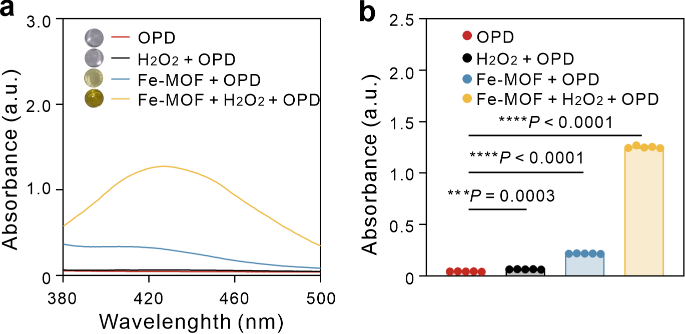


**Figure S11.** (a, b) UV-vis absorption spectra of OPD (a) and corresponding absorption intensity (b) at 430 nm, n = 3. Statistical significance denoted as ***P < 0.001 and ****P < 0.0001, analyzed by one-way ANOVA, followed by Dunnett’s multiple comparisons test. Data represent mean ± s.d.


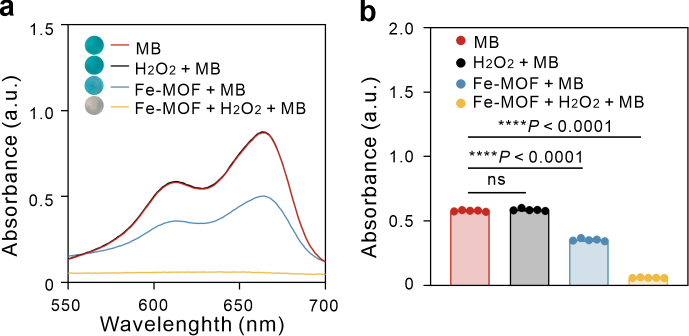


**Figure S12**. (a, b) UV-vis absorption spectra of MB (a) and corresponding intensities (b) at 1617 nm treated with various treatments, n = 3. Statistical significance denoted as *P < 0.05, ****P < 0.0001, and ns: not significant (P > 0.05), analyzed by one-way ANOVA, followed by Dunnett’s multiple comparisons test. Data represent mean ± s.d.


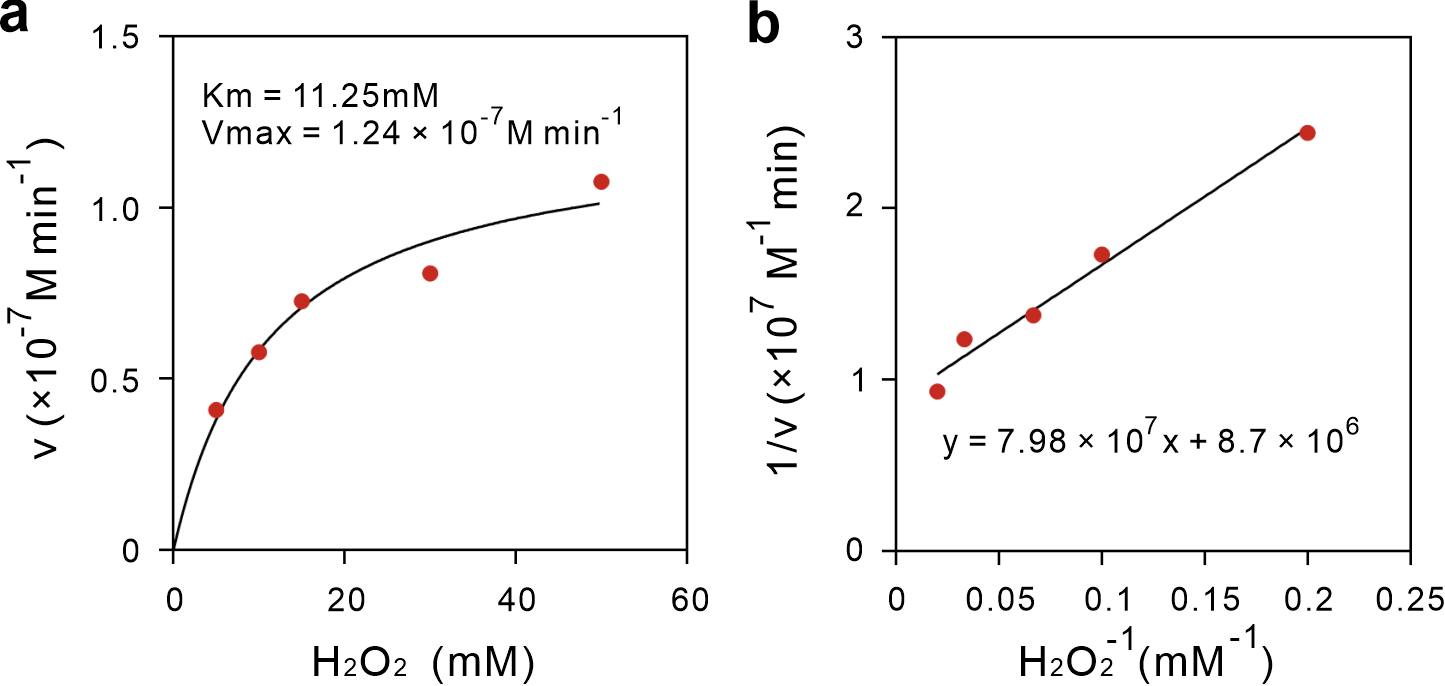


**Figure S13**. (a, b) The Michaelis-Menten kinetic analysis (a) and Linweaver-Burk plot for Fe-MOF with H_2_O_2_ as a substrate (b).


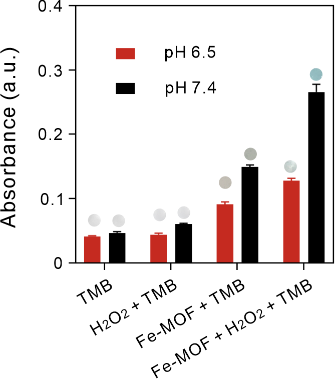


**Figure S14**. The absorbance of oxTMB at 650nm under different pH values, n = 3. Statistical significance denoted as *P < 0.05, ****P < 0.0001, and ns: not significant (P > 0.05), analyzed by one-way ANOVA, followed by Dunnett’s multiple comparisons test. Data represent mean ± s.d.


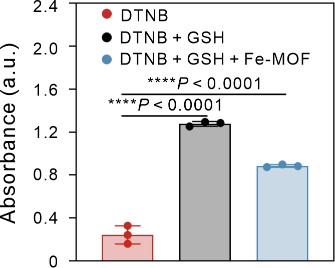


**Figure S15**. UV-vis corresponding absorbance at 450 nm of DTNB solutions after reactions, n = 3. Statistical significance denoted as ****P < 0.0001, analyzed by one-way ANOVA, followed by Dunnett’s multiple comparisons test. Data represent mean ± s.d.


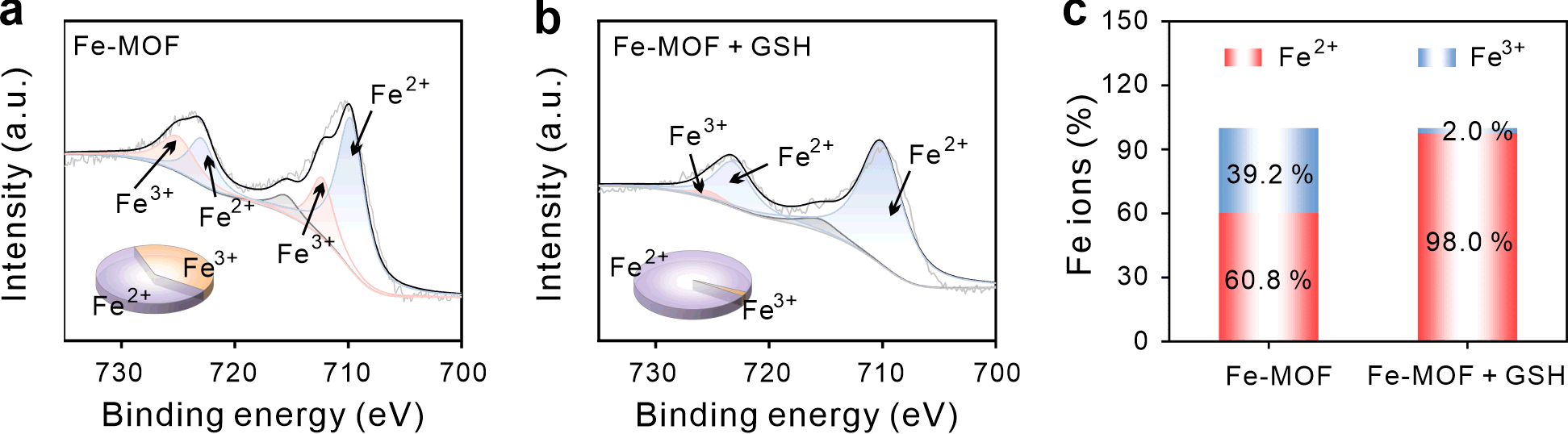


**Figure S16.** (a, b) High-resolution Fe 2p spectra of Fe-MOF (a) and Fe-MOF + GSH (b). Insert: Percentage of Fe^2+^ and Fe^3+^ in Fe-MOF. (c) Valence state changes of Fe-MOF after reaction with 10 mM GSH for 12 h.


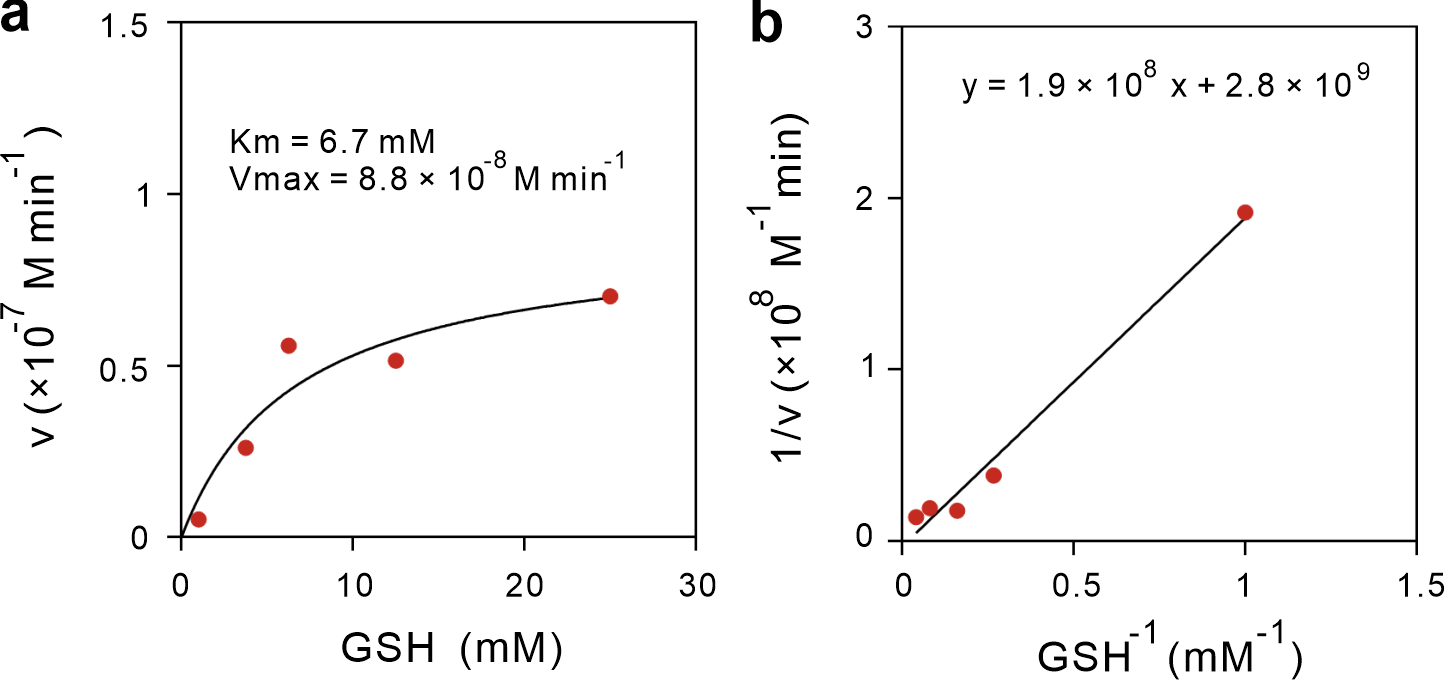


**Figure S17**. (a, b) The Michaelis-Menten kinetic analysis(a) and Linweaver-Burk plot of GSHox-like properties of Fe-MOF (b).


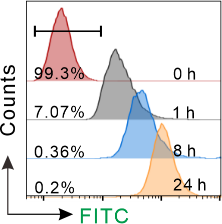


**Figure S18.** Flow cytometry analysis of cellular uptake of FITC-labeled Fe-MOF/P at different time points.


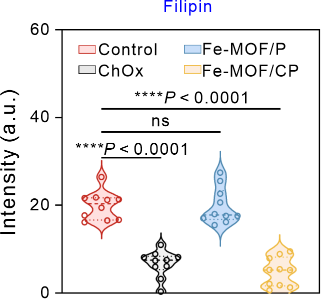


**Figure S19**. The fluorescence intensities of 4T1 cells incubation with Filipin complex, n = 10. Statistical significance denoted as ****P < 0.0001 and ns: not significant (P > 0.05), analyzed by one-way ANOVA, followed by Dunnett’s multiple comparisons test. Data represent mean ± s.d.


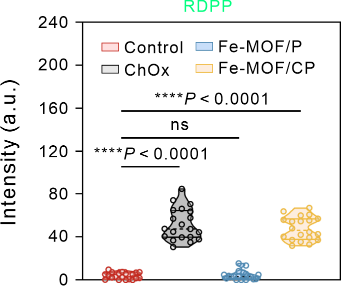


**Figure S20**. Quantitative analysis of fluorescent intensity of O_2_ probe by Image J software, n = 20. Statistical significance denoted as ****P < 0.0001 and ns: not significant (P > 0.05), analyzed by one-way ANOVA, followed by Dunnett’s multiple comparisons test. Data represent mean ± s.d.


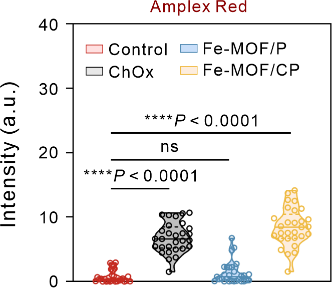


**Figure S21.** Statistical analysis of fluorescence intensity of H_2_O_2_, n = 29. Statistical significance denoted as ****P < 0.0001 and ns: not significant (P > 0.05), analyzed by one-way ANOVA, followed by Dunnett’s multiple comparisons test. Data represent mean ± s.d.


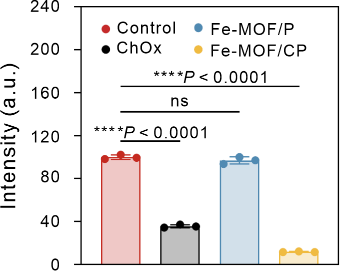


**Figure S22**. The relative intensity of western blot results for CD59 expression levels in different experimental groups, n = 3. Statistical significance denoted as ****P < 0.0001 and ns: not significant (P > 0.05), analyzed by one-way ANOVA, followed by Dunnett’s multiple comparisons test. Data represent mean ± s.d.


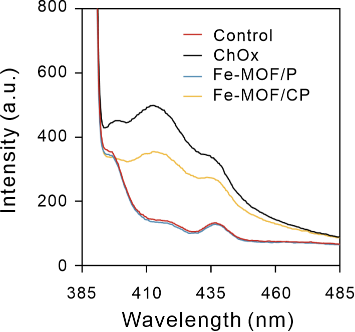


**Figure S23**. The fluorescence spectra of 4T1cells stained with 1-pyrenedodecanoic acid.


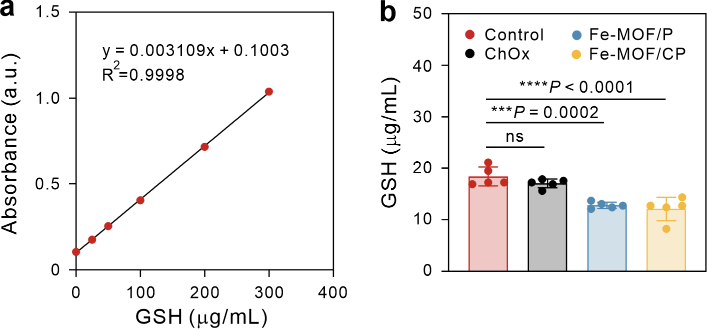


**Figure S24**. (a, b) The standard cure (a) and concentration (b) of GSH in 4T1 cells, n = 5. Statistical significance denoted as ***P < 0.001, ****P < 0.0001 and ns: not significant (P > 0.05), analyzed by one-way ANOVA, followed by Dunnett’s multiple comparisons test. Data represent mean ± s.d.


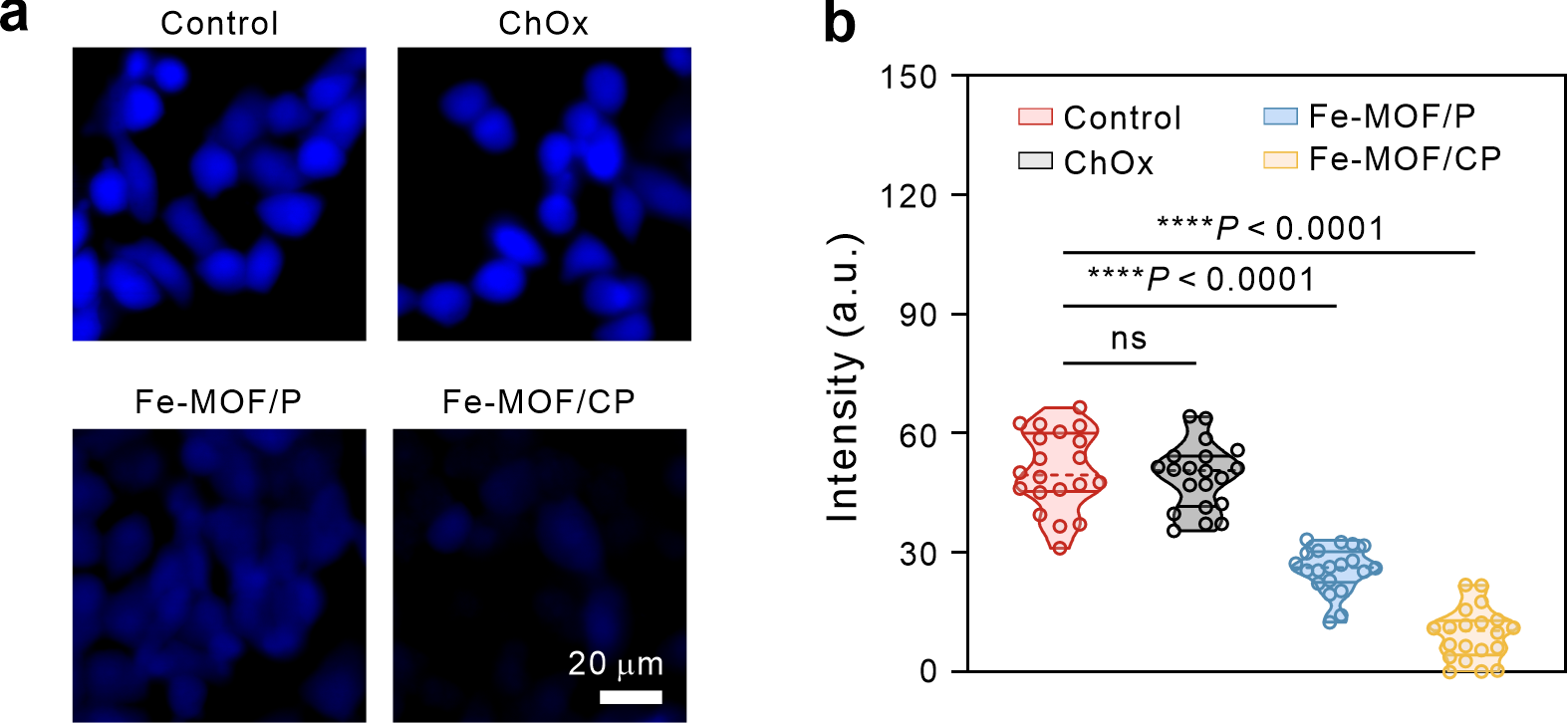


**Figure S25.** (a, b) Cellular fluorescent images of 4T1 cells after receiving the indicated treatments and staining with Bromodiamine (a) and statistical analysis of Bromodiamine fluorescence intensity (b), n = 20. Statistical significance denoted as ****P < 0.0001 and ns: not significant (P > 0.05), analyzed by one-way ANOVA, followed by Dunnett’s multiple comparisons test. Data represent mean ± s.d.


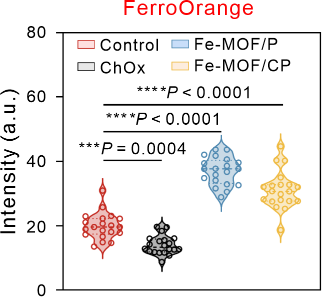


**Figure S26**. Statistical analysis of fluorescent intensity of Fe^2+^, n = 20. Statistical significance denoted as ***P < 0.001 and ****P < 0.0001, analyzed by one-way ANOVA, followed by Dunnett’s multiple comparisons test. Data represent mean ± s.d.


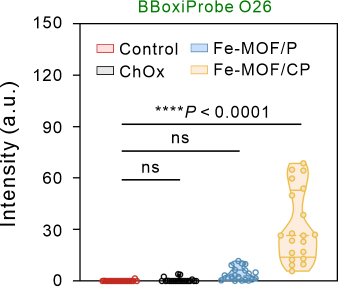


**Figure S27.** Statistical analysis of fluorescence intensity of ^•^OH, n = 20. Statistical significance denoted as ****P < 0.0001 and ns: not significant (P > 0.05), analyzed by one-way ANOVA, followed by Dunnett’s multiple comparisons test. Data represent mean ± s.d.


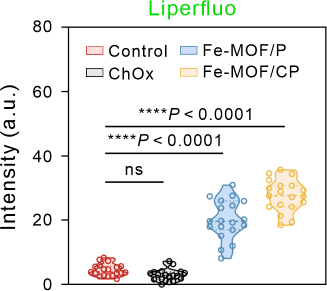


**Figure S28.** Statistical analysis of fluorescence intensity of Liperfluo, n = 19. Statistical significance denoted as ****P < 0.0001 and ns: not significant (P > 0.05), analyzed by one-way ANOVA, followed by Dunnett’s multiple comparisons test. Data represent mean ± s.d


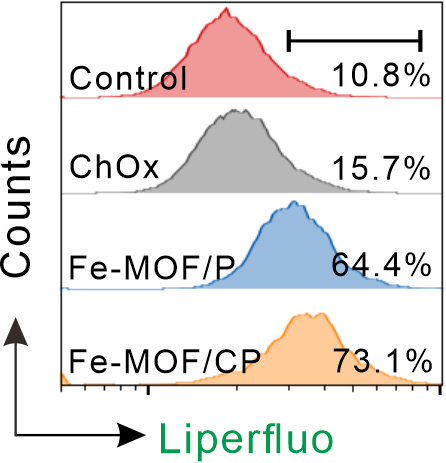


**Figure S29**. Representative flow cytometric results of 4T1 cells stained with Liperfluo.


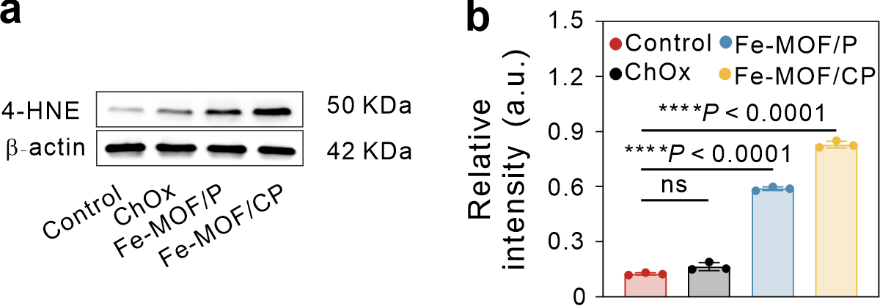


**Figure S30**. (a, b) Western blotting results (a) and relative intensity (b) of 4-HNE expression in different experimental groups, n = 3. Statistical significance denoted as ****P < 0.0001 and ns: not significant (P > 0.05), analyzed by one-way ANOVA, followed by Dunnett’s multiple comparisons test. Data represent mean ± s.d.


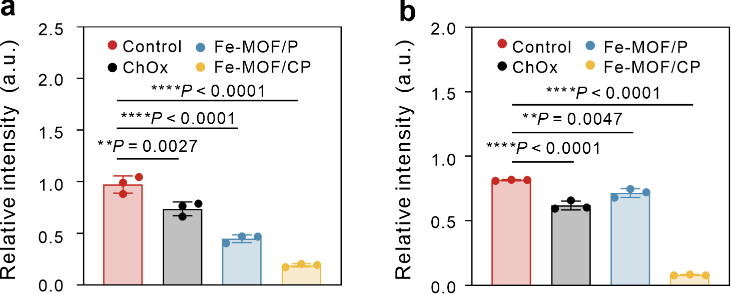


**Figure S31**. (a, b) The relative intensity of western blot results for GPX4 (a) and FSP1 (b) expression levels in different experimental groups, n = 3. Statistical significance denoted as **P < 0.01, ****P < 0.0001 and ns: not significant (P > 0.05), analyzed by one-way ANOVA, followed by Dunnett’s multiple comparisons test. Data represent mean ± s.d


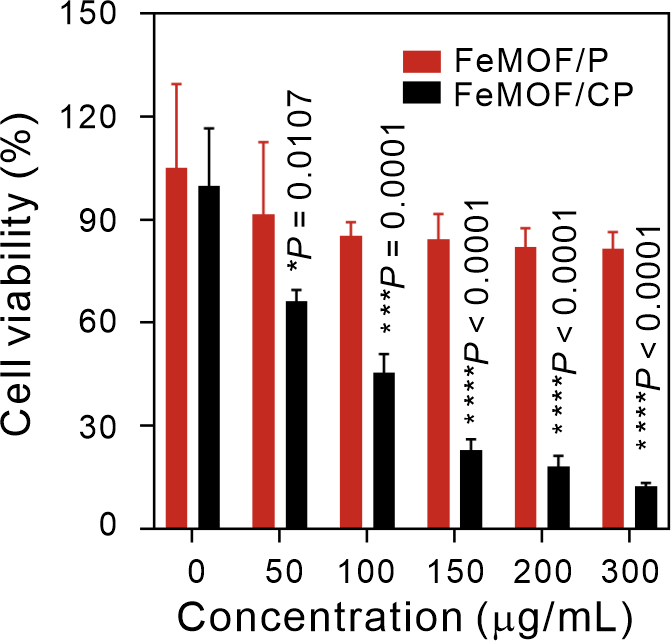


**Figure S32.** The viability of 4T1 cells treated with various concentrations of Fe-MOF/P and Fe-MOF/CP for 24 h, n = 3. Statistical significance denoted as **P < 0.01 and ****P < 0.0001, analyzed by two-way ANOVA, followed by Dunnett’s multiple comparisons test. Data represent mean ± s.


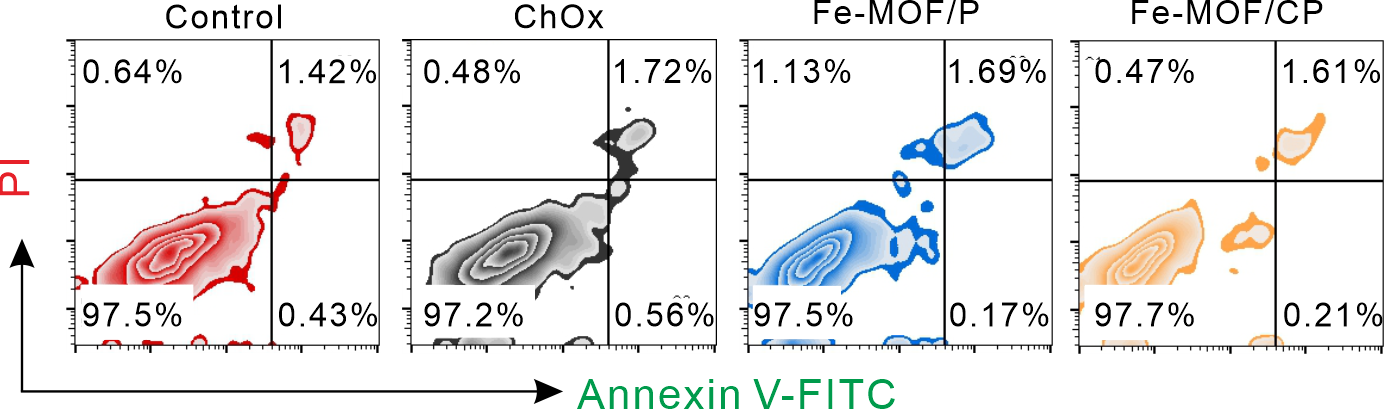


**Figure S33**. The apoptosis assay results of CTLL2 cells received indicated treatments.


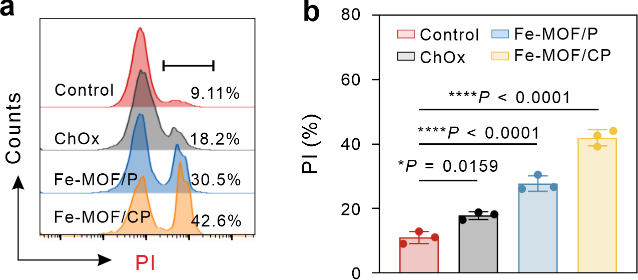


**Figure S34**. (a, b) Apoptosis rates (a) and quantitative analysis (b) of 4T1 cells under different treatments by flow cytometry, n = 3. Statistical significance denoted as *P <0.5, ****P < 0.0001 and ns: not significant (P > 0.05), analyzed by one-way ANOVA, followed by Dunnett’s multiple comparisons test. Data represent mean ± s.d.


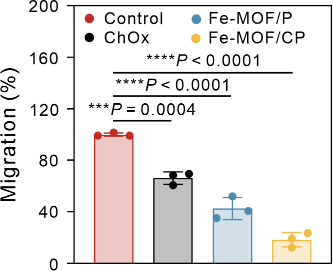


**Figure S35**. Statistical analysis of migration rates, n = 3. Statistical significance denoted as ***P < 0.001 and ****P < 0.0001, analyzed by one-way ANOVA, followed by Dunnett’s multiple comparisons test. Data represent mean ± s.d.


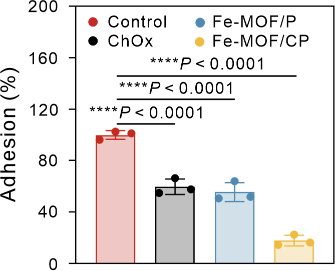


**Figure S36**. Statistical analysis of adhesion percentage, n = 3. Statistical significance denoted as ****P < 0.0001, analyzed by one-way ANOVA, followed by Dunnett’s multiple comparisons test. Data represent mean ± s.d.


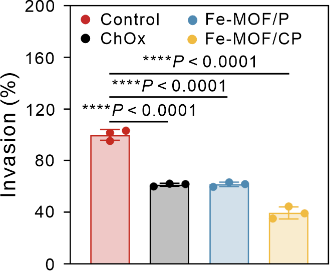


**Figure S37**. Statistical analysis of invasion percentages, n = 3. Statistical significance denoted as ****P < 0.0001, analyzed by one-way ANOVA, followed by Dunnett’s multiple comparisons test. Data represent mean ± s.d.


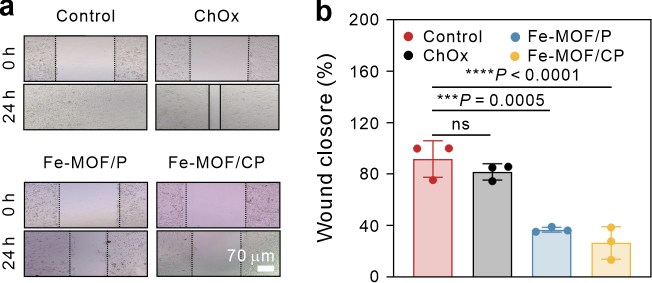


**Figure S38.** (a, b) Images (a) and statistical analysis (b) of wound healing of 4T1 cells with diverse treatments, n = 3. Scale bar: 70 μm. Statistical significance denoted as ***P < 0.001, ****P < 0.0001, and ns: not significant (P > 0.05), analyzed by one-way ANOVA, followed by Dunnett’s multiple comparisons test. Data represent mean ± s.d.
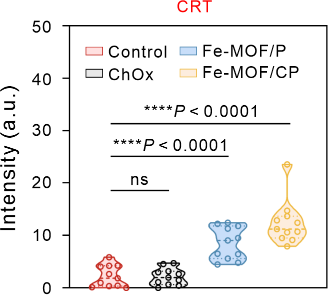


**Figure S39**. CRT intensity of 4T1 cells after indicated treatments, n = 11. Statistical significance denoted as ****P < 0.0001 and ns: not significant (P > 0.05), analyzed by one-way ANOVA, followed by Dunnett’s multiple comparisons test. Data represent mean ± s.d.


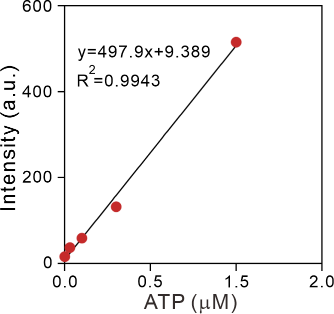


**Figure S40**. Standard curve of ATP.


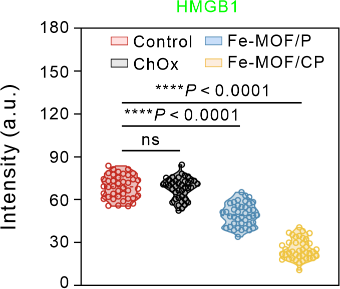


**Figure S41**. HMGB1 immunofluorescence statistical results of 4T1 cells after indicated treatments, n = 40. Statistical significance denoted as ****P < 0.0001 and ns: not significant (P > 0.05), analyzed by one-way ANOVA, followed by Dunnett’s multiple comparisons test. Data represent mean ± s.d.


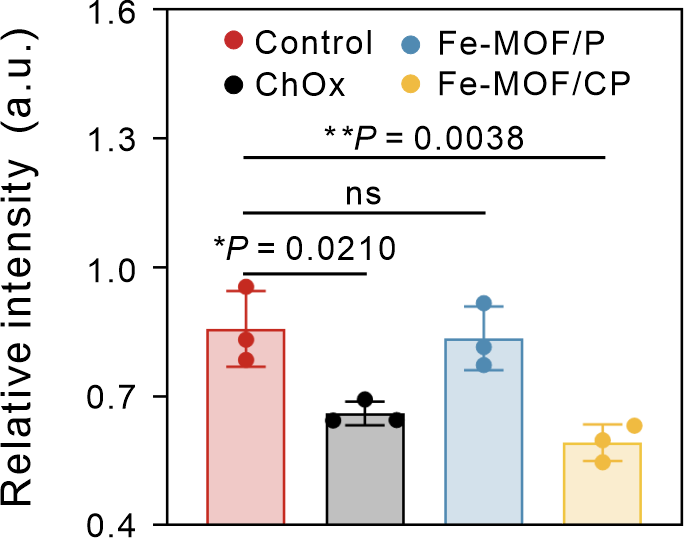


**Figure** **S42**. The western blot intensity statistics for PD-L1 expression levels after different treatments, n = 3. Statistical significance denoted as ****P < 0.0001 and ns: not significant (P > 0.05), analyzed by one-way ANOVA, followed by Dunnett’s multiple comparisons test. Data represent mean ± s.d.


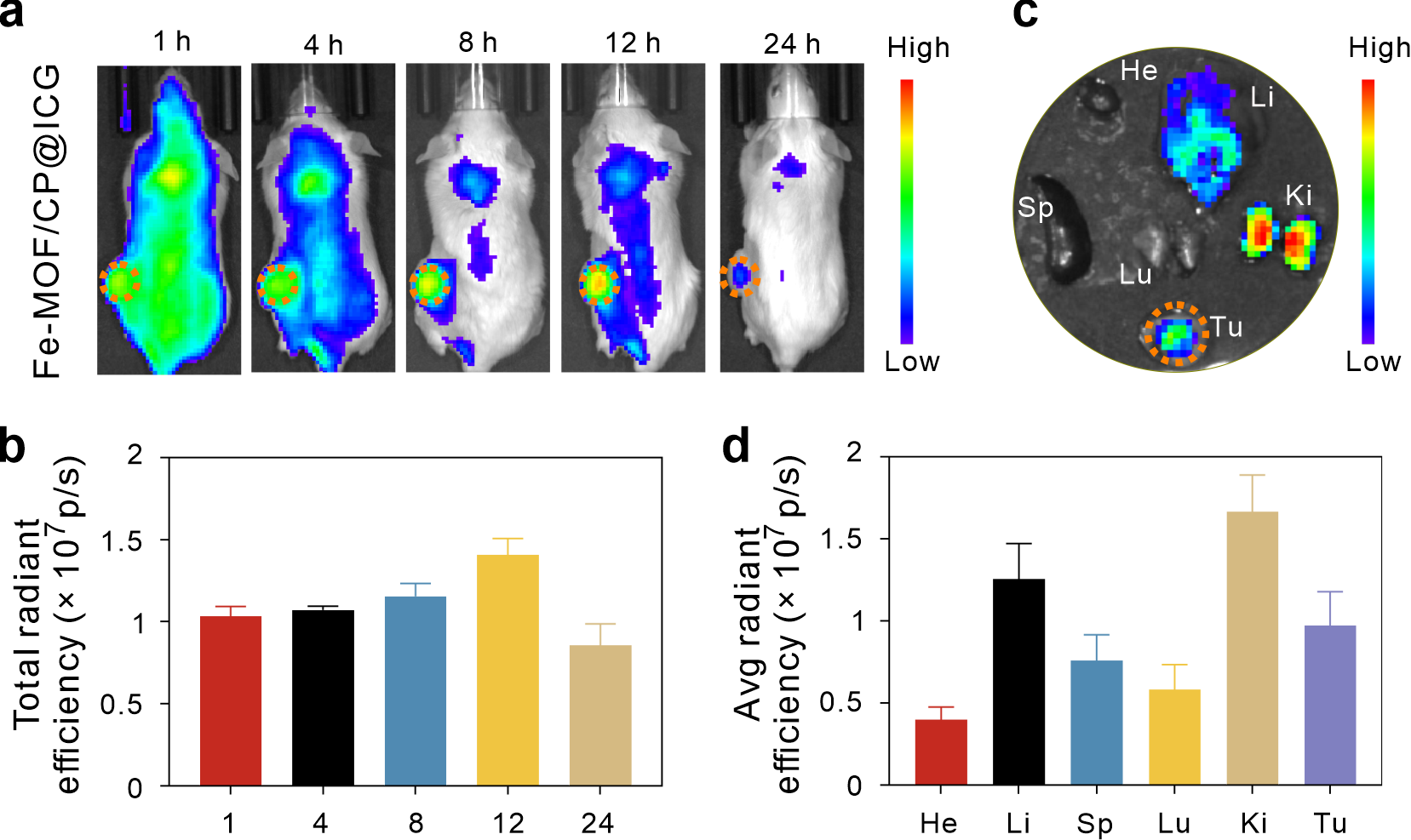


**Figure S43.** (a, b) Representative fluorescent images of mice receiving i. v. injection of 5 mg/kg Fe-MOF/CP@ICG at various time points (a) and the corresponding statistical analysis of fluorescent intensity (b) in the tumor regions. (c, d) *Ex vivo* images of the major organs from mice 24 h post-injection (c) and the corresponding statistical analysis of fluorescent intensity in these organs (d).


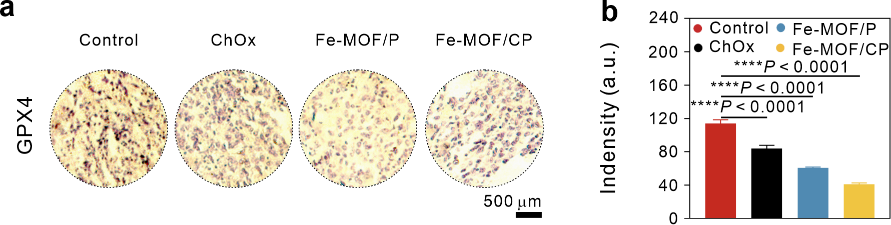


**Figure S44**. (a, b) IHC staining images of GPX4 (a) and corresponding intensities (b), n = 3. Statistical significance denoted as ****P < 0.0001 , analyzed by one-way ANOVA, followed by Dunnett’s multiple comparisons test. Data represent mean ± s.d.


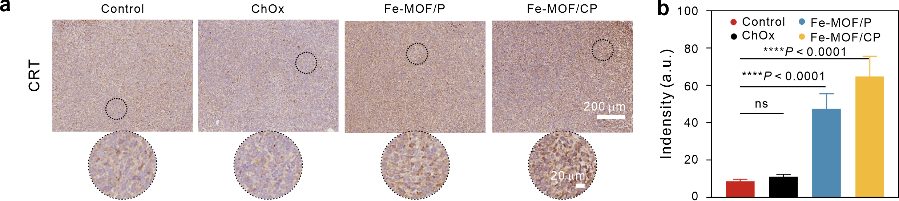


**Figure S45**. (a, b) IHC staining images of CRT (a) and corresponding intensities (b). n = 3. Statistical significance denoted as ****P < 0.0001 and ns: not significant (P > 0.05), analyzed by one-way ANOVA, followed by Dunnett’s multiple comparisons test. Data represent mean ± s.d.


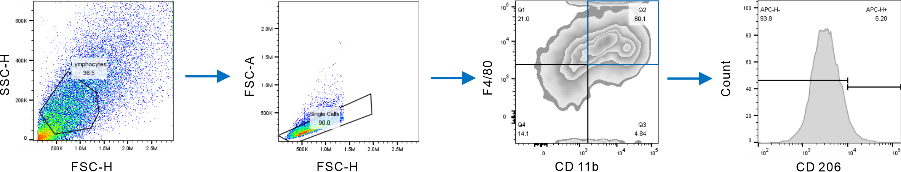


**Figure S46**. Gating strategy for flow cytometry of macrophages in tumors.


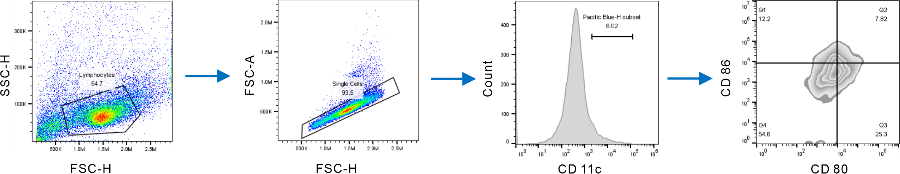


**Figure S47**. Gating strategy for flow cytometry of DC cells in lymph nodes.


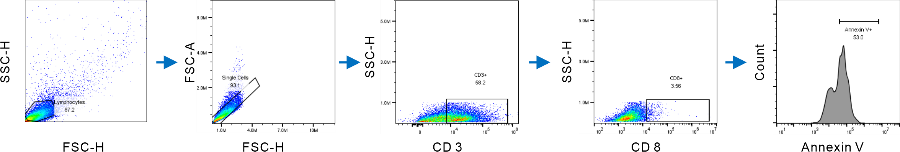


**Figure S48**. Gating strategy for flow cytometry of CD8^+^ cells exhaustion in tumors.


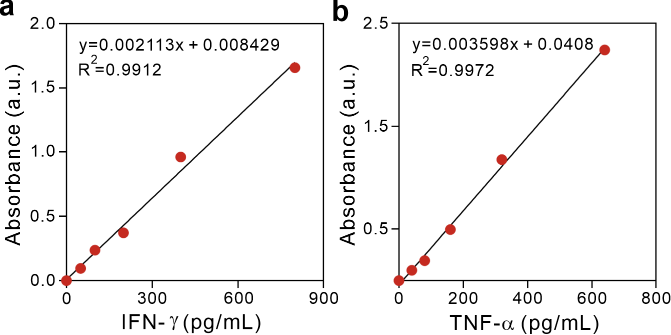


**Figure S49**. (a, b) Standard curves of IFN-γ (a) and TNF-α (b).


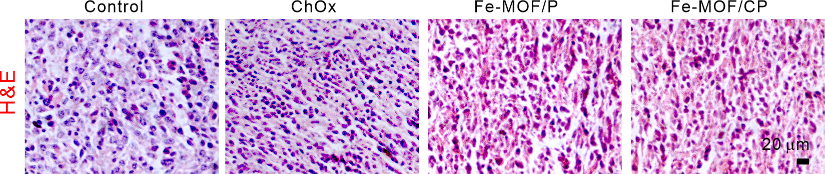


**Figure S50**. H&E staining of 4T1 tumors treated with PBS, ChOx, Fe-MOF/P, or Fe-MOF/CP, Scale bar: 20 μm.


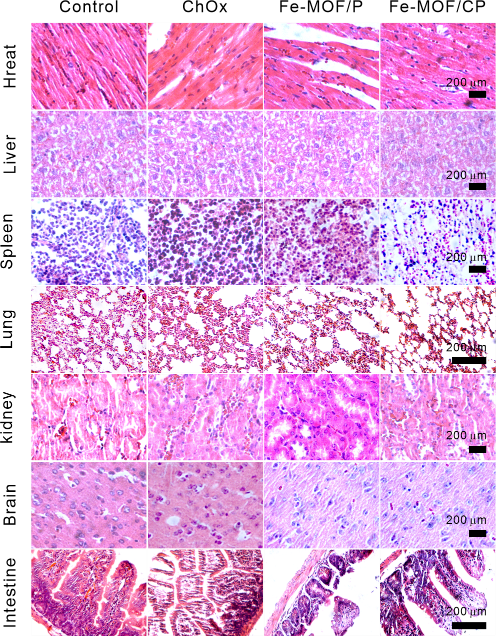


**Figure S51**. H&E staining of the major organs after treatments, scale bars: 200 µm.


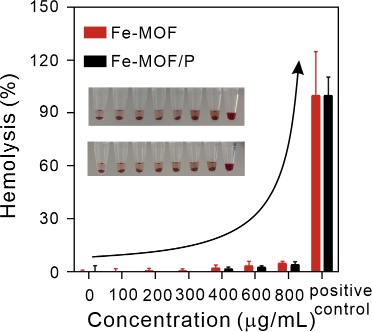


**Figure S52.** Hemolytic percentages of red blood cells incubated with Fe-MOF or Fe-MOF/P at the concentrations of 0, 100, 200, 300, 400, 600, and 800 µg mL^−1^ for 2 h, n = 3. Statistical significance denoted as ****P < 0.0001 and ns: not significant (P > 0.05), analyzed by one-way ANOVA, followed by Dunnett’s multiple comparisons test. Data represent mean ± s.d.


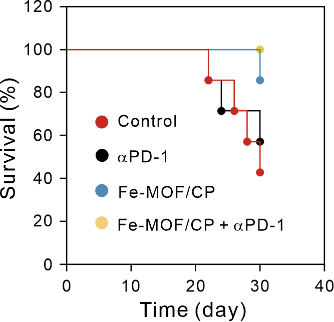


**Figure S53**. Survival percentage of mice after different treatments in two-sided model, n = 7.


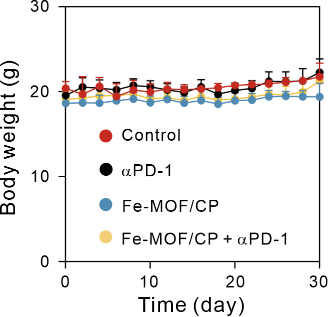


**Figure S54**. Body weights of mice after different treatments in two-sided model, n = 7.


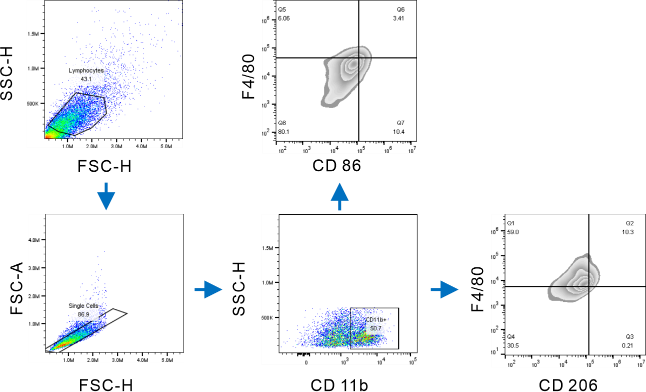


**Figure S55.** Gating strategy for flow cytometry of macrophages in tumors.


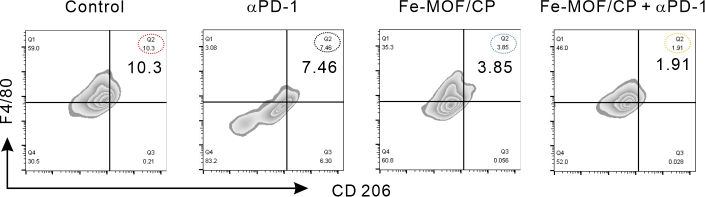


**Figure S56.** The representative flow cytometric results (gated on CD11b^+^F4/80^+^CD206^+^) in tumors.


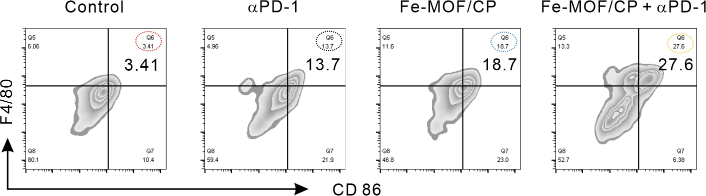


**Figure S57.** The representative flow cytometric results (gated on CD11b^+^F4/80^+^CD86^+^) in tumors.


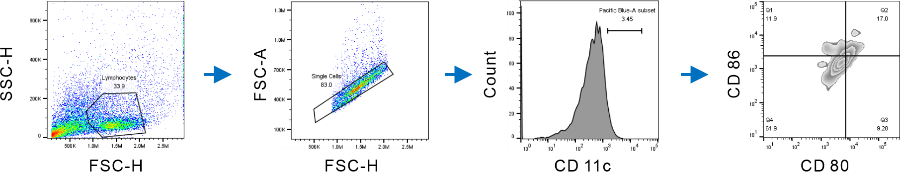


**Figure S58.** Gating strategy for flow cytometry of DC maturation in lymph gland.


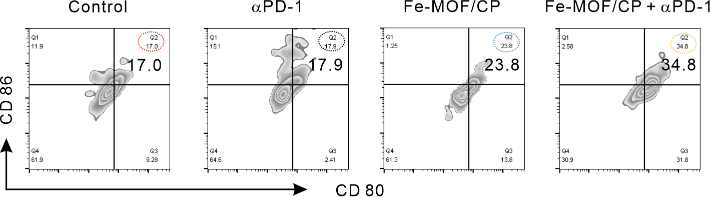


**Figure S59.** The representative flow cytometric results gated on (CD11c^+^CD80^+^CD86^+^) in lymph gland.


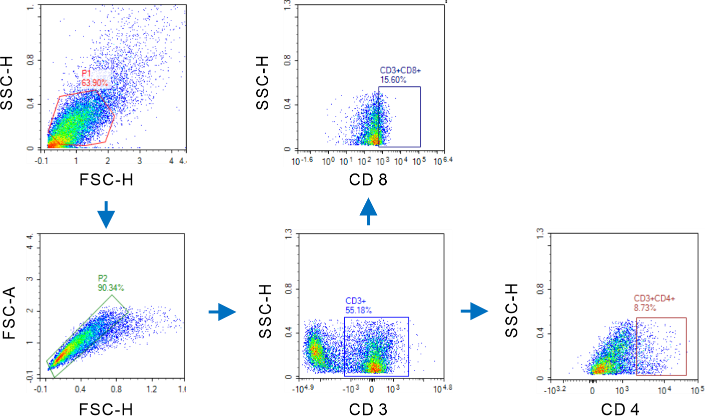


**Figure S60**. Gating strategy for flow cytometry of T cells in tumors.


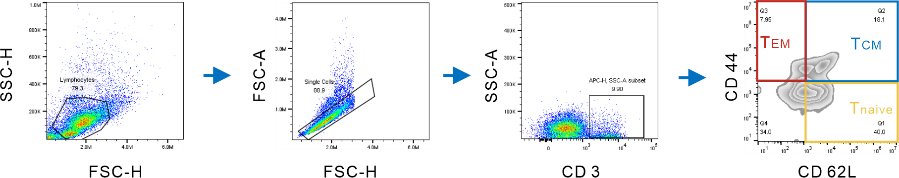


**Figure S61**. Gating strategy for flow cytometry of memory T cells in spleens.


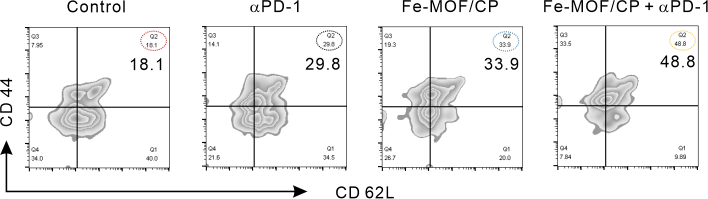


**Figure S62**. The representative flow cytometric results of memory T cells in spleens.

**Table S1**. The absorbance measurements, calculated concentrations, and resulting loading rates of ChOx for various groups (total and supernatant) as illustrated in Figure S5. Data are presented as mean (M) ± standard deviation (SD), n = 3.

| Groups | Abs (a.u.) | ChOx (μg/mL) | Loading rate (%) |
| --- | --- | --- | --- |
|  | M ± SD | M ± SD | M ± SD |
| 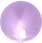Total | 0.278 ± 0.013 | 94.79 ± 6.9  .9 | 62.8 ±1.27 |
| 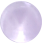Supernatant | 0.166 ± 0.006 | 35.28 ± 3.3  3.3 |  |

**Table S2**. The brand, clone, and catalog number of all antibodies used in flow cytometry.

| Antibodies | Brand | Clone | Catalog number |
| --- | --- | --- | --- |
| PE/Cyanine7 anti-mouse CD3 | Biolegend | 17A2 | 100219 |
| PerCP-Cy^TM^5.5 anti-mouse CD8a | BD biosciences | 53-6.7 | 551162 |
| APC/Cyanine7 anti-mouse/human CD11b | Biolegend | M1/70 | 101226 |
| PE-CF594 anti-mouse F4/80 | BD biosciences | T45-2342 | 565613 |
| FITC-Annexin V | Biolegend | / | 640906 |
| Alexa Fluor™ 647 Rat anti-Mouse CD206 | BD biosciences | Y17-505 | 568809 |
| BV421 anti-mouse CD11c | BD biosciences | N418 | 565451 |
| FITC anti-mouse CD80 | Biolegend | 16-10A1 | 104706 |
| PE anti-mouse CD86 | Biolegend | GL-1 | 105008 |
| APC anti-mouse CD86 | Biolegend | GL-1 | 105011 |
| FITC anti-mouse CD206 (MMR) | Biolegend | C068C2 | 141703 |
| Brilliant Violet 421™ anti-mouse CD4 | Biolegend | GK1.5 | 100443 |
| APC/Cyanine7 anti-mouse CD8a | Biolegend | 53-6.7 | 100714 |
| APC anti-mouse CD3 | Biolegend | 17A2 | 100236 |
| FITC anti-mouse/human CD44 | Biolegend | IM7 | 103005 |
| BV650 anti-mouse CD62L | BD biosciences | MEL-14 | 564108 |
